# Loss of *Cpt1a* results in elevated glucose-fueled mitochondrial oxidative phosphorylation and defective hematopoietic stem cells

**DOI:** 10.1172/JCI184069

**Published:** 2025-01-09

**Authors:** Jue Li, Jie Bai, Vincent T. Pham, Michihiro Hashimoto, Maiko Sezaki, Qili Shi, Qiushi Jin, Chenhui He, Amy Armstrong, Tian Li, Mingzhe Pan, Shujun Liu, Yu Luan, Hui Zeng, Paul R. Andreassen, Gang Huang

**Affiliations:** 1 Department of Hematology, Xiangya Hospital, Central South University, Changsha, Hunan, China.; 2 Experimental Hematology and Cancer Biology, Cincinnati Children’s Hospital Medical Center (CCHMC), Cincinnati, Ohio, USA.; 3 Department of Cell Systems and Anatomy, UT Health San Antonio, Joe R. and Teresa Lozano Long School of Medicine, San Antonio, Texas, USA.; 4 Mays Cancer Center at UT Health San Antonio, San Antonio, Texas, USA.; 5 Department of Pathology and Laboratory Medicine, UT Health San Antonio, Joe R. and Teresa Lozano Long School of Medicine, San Antonio, Texas, USA.; 6 Department of Microbiology, Immunology and Molecular Genetics, UT Health San Antonio, Joe R. and Teresa Lozano Long School of Medicine, San Antonio, Texas, USA.; 7 Department of Medicine, The MetroHealth System, Case Western Reserve University, Cleveland, Ohio, USA.; 8 Greehey Children’s Cancer Research Institute, University of Texas Health Science Center at San Antonio, San Antonio, Texas, USA.; 9 Department of Hematology, Guangdong Provincial People’s Hospital (Guangdong Academy of Medical Sciences), Southern Medical University, Guangzhou, Guangdong, China.; 10 Department of Pediatrics, University of Cincinnati College of Medicine, Cincinnati, Ohio, USA.

**Keywords:** Hematology, Metabolism, Hematopoietic stem cells

## Abstract

Hematopoietic stem cells (HSCs) rely on self-renewal to sustain stem cell potential and undergo differentiation to generate mature blood cells. Mitochondrial fatty acid β-oxidation (FAO) is essential for HSC maintenance. However, the role of carnitine palmitoyl transferase 1a (CPT1A), a key enzyme in FAO, remains unclear in HSCs. Using a *Cpt1a* hematopoiesis-specific conditional-KO (*Cpt1a*^Δ*/*Δ^) mouse model, we found that loss of *Cpt1a* led to HSC defects, including loss of HSC quiescence and self-renewal and increased differentiation. Mechanistically, we found that loss of *Cpt1a* resulted in elevated levels of mitochondrial respiratory chain complex components and their activity, as well as increased ATP production and accumulation of mitochondrial ROS in HSCs. Taken together, this suggests hyperactivation of mitochondria and metabolic rewiring via upregulated glucose-fueled oxidative phosphorylation (OXPHOS). In summary, our findings demonstrate an essential role for *Cpt1a* in HSC maintenance and provide insight into the regulation of mitochondrial metabolism via control of the balance between FAO and glucose-fueled OXPHOS.

## Abstract 2

Cpt1a’s key role in HSC maintenance and its regulation of mitochondrial metabolism by balancing FAO and glucose-fueled OXPHOS.

## Graphical abstract

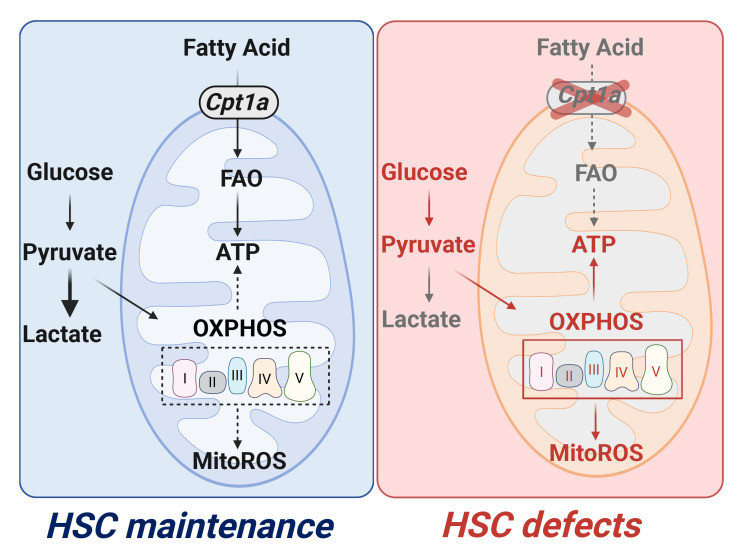

## Introduction

Hematopoietic stem cells (HSCs), a specialized cell population predominantly located in the bone marrow (BM) niche of humans and mice, play a vital role in sustaining lifelong hematopoiesis (1, 2). Long-term hematopoietic stem cells (LT-HSCs) represent a rare subset of this cell population, the majority of which maintain a state of quiescence under steady-state conditions to preserve their potential for self-renewal and differentiation (3, 4). However, the mechanisms involved in HSC self-renewal and differentiation, and their regulation, are not well understood.

Mitochondrial fatty acid β-oxidation (FAO) plays a crucial role in supporting the maintenance of HSCs by mechanisms that have not been clearly defined (5–8). Previously, inhibition of mitochondrial FAO has mainly been achieved by inhibiting the rate-limiting enzyme carnitine palmitoyl transferase 1a (CPT1A) (5, 9, 10). In particular, *Cpt1a*-dependent FAO has been identified as crucial for the survival of embryonic stem cells and maintenance of neural stem/progenitor cell (11, 12). However, the specific role of *Cpt1a* in the regulation of HSC maintenance is still unclear.

CPT1A, located in the mitochondrial outer membrane, facilitates the transport of fatty acids into mitochondria, where they are broken down via the FAO pathway, thereby leading to the production of ATP (13). Specifically, the CPT1A-mediated FAO pathway generates NADH, which can be used later by mitochondrial respiratory chain complexes to produce ATP through oxidative phosphorylation (OXPHOS). Furthermore, acetyl-CoA, an end product of FAO, can enter the TCA, where both NADH and ATP are also generated. Recent studies have highlighted the important role of mitochondrial metabolism in regulating both the self-renewal and differentiation capacities of HSCs (14–16). It is generally believed that a metabolic transition of HSCs from glycolysis to OXPHOS and the increase of ROS drive the exit of HSCs from quiescence and initiate their subsequent differentiation (17). However, the precise role of *Cpt1a* and FAO in HSCs, and of mitochondrial metabolism more generally, remains elusive.

In this study, using *Cpt1a* hematopoiesis-specific conditional-KO (*Cpt1a*^Δ*/*Δ^) mice, we demonstrate that deletion of *Cpt1a* led to HSC defects, impaired quiescence and self-renewal, and enhanced differentiation. This was mediated through hyperactivation of mitochondrial OXPHOS, increased respiratory chain complexes, and elevated OXPHOS byproducts such as mitochondrial ROS (mtROS). These findings underscore the critical role of *Cpt1a* and FAO in regulating HSC maintenance. Additionally, we propose a mechanistic role for *Cpt1a* in the regulation of HSC self-renewal and differentiation by controlling the balance between FAO and OXPHOS and, as a consequence, the state of mitochondrial metabolism.

## Results

### Cpt1a is enriched in HSCs, and Cpt1a^Δ/Δ^ impairs HSCs and promotes differentiation.

**Figure 1:**
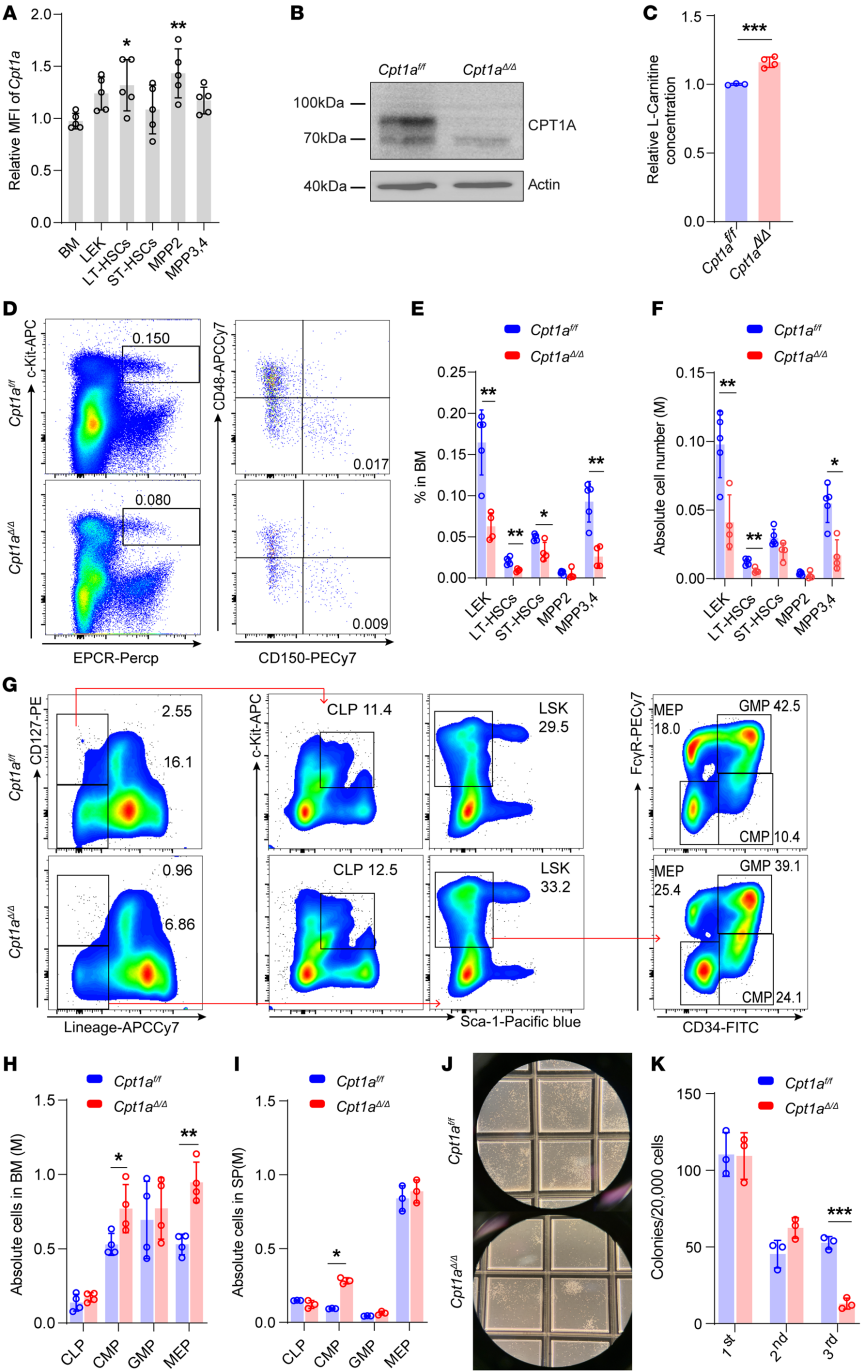
*Cpt1a* is enriched in HSCs, and *Cpt1a^Δ/Δ^* impairs HSCs and promotes differentiation. (**A**) Relative expression of CPT1A evaluated by flow cytometry in different hematopoietic cell subsets. Each subset was compared with total BM cells for statistical analyses. LEK cells: Lin^–^EPCR^+^c-Kit^+^; LT-HSCs: CD150^+^CD48^–^LEK; ST-HSCs: CD150^–^CD48^–^ LEK; MPP2 cells: CD150^+^CD48^+^ LEK; MPP3,4 cells: CD150^–^CD48^+^ LEK. *n* = 5 per group. (**B**) Western blot analysis was performed to determine the expression of CPT1A in c-Kit^+^ cells. Results are representative of 3 independent experiments. (**C**) Relative l-carnitine concentration measured by ELISA in c-Kit^+^ cells. *n* = 3 or 4. (**D**) Representative flow cytometric analysis of HSC populations gated from the Lin^–^ cell population in the BM. HSC populations were as in **A**. (**E** and **F**) Frequencies and absolute numbers of the indicated BM populations. M, million. *n* = 5 or 4. (**G**) Representative flow cytometric analysis of HSPC populations in the BM. CMPs: CD34^+^FcγR^–^LK; granulocyte/monocyte progenitors (GMPs): CD34^+^FcγR^+^ LK; MEPs: CD34^–^FcγR^–^LK; common lymphoid progenitors (CLPs): CD127^+^c-Kit^lo^Sca-1^lo^Lin^–^. (**H** and **I**) Absolute numbers of HSPC populations in the BM and spleen. *n* = 4 or 3. (**J**) CFU cell colonies. Original magnification, ×40. (**K**) Statistics for CFU colonies over 3 serial replatings. *n* = 3 per group. Data represent the mean ± SEM. *P < 0.05, **P < 0.01, and ***P < 0.001, by Student’s *t* test.

Proper HSC function is dependent on the metabolic state, and CPT1A transports fatty acids into mitochondria, where they can be metabolized via FAO (18, 19). To investigate the role of *Cpt1a* in HSCs, we first identified high expression levels of *Cpt1a* in both human and mouse HSCs, especially long-term HSCs (LT-HSCs) (Supplemental Figure 1E), using a publicly available database (Supplemental Figure 1, A–D; supplemental material available online with this article; https://doi.org/10.1172/JCI184069DS1). We confirmed CPT1A expression in WT mouse BM cells utilizing an endothelial protein C receptor–based (EPCR-based) fraction to identify HSCs without relying on the traditional marker Stem cell antigen-1 (Sca-1). Previous studies have shown that this method accurately identifies HSCs (20–23). It has been reported that the EPCR marker surpasses the Sca-1 marker in labeling HSCs, as it consistently identifies stem cells both before and after 5-fluorouracil (5-FU) treatment (22). Notably, *Cpt1a* manifested heightened expression in LT-HSCs and multipotent progenitor 2 (MPP2) cells, as compared with whole BM cells (Figure 1A).

To understand the role of CPT1A in hematopoiesis, we first treated WT mice with the CPT1A inhibitor etomoxir for varying durations and observed a decrease in LT-HSC frequencies and numbers over time. Furthermore, the proportion of cells in the G_0_ phase increased, while BM cell numbers remained comparable (Supplemental Figure 2, A–D). Although we noted changes in HSC maintenance, it is important to note that etomoxir has been reported to have off-target effects in vivo (24) and had time-dependent effects in our in vivo experiments.

As an additional avenue to understand the specific role of *Cpt1a* in LT-HSCs and hematopoiesis, we crossed *Cpt1a^fl/fl^* mice with *Vav1-Cre^+^* mice, resulting in targeted deletion of the *Cpt1a* gene within the hematopoietic system (Supplemental Figure 3A). PCRs (Supplemental Figure 3B) and Western blotting (Figure 1B) were used to confirm the successful KO of the *Cpt1a* gene and its expression in c-Kit^+^ BM cells. As a lack of CPT1A activity leads to failure to generate acylcarnitine, which enters the mitochondria for oxidation (18), we found that l-carnitine concentrations were higher in *Cpt1a*^Δ*/*Δ^ HSCs (Figure 1C). *Cpt1a*^Δ*/*Δ^ mice had phenotypes comparable to those of *Cpt1a^fl/fl^* mice, including similar body, spleen, and liver weights, as well as similar BM and splenic cell counts and complete blood counts (Supplemental Figure 4, A–I). We found that platelet counts slightly decreased in *Cpt1a*^Δ*/*Δ^ mice (Supplemental Figure 4J). Interestingly, *Cpt1a*^Δ*/*Δ^ mice exhibited a substantial reduction in the frequencies of lineage^–^EPCR^+^c-Kit^+^ (LEK) cells, LT-HSCs, short-term HSCs (ST-HSCs), and MPP3 and MPP4 (MPP3,4) cells, and showed reduced absolute numbers of LEK cells, LT-HSCs, and MPP3,4 populations (Figure 1, D–F). We further analyzed hematopoietic progenitor cells (HPCs) in the BM and spleen (Figure 1G and Supplemental Figure 5, A and B). The absolute numbers of common myeloid progenitors (CMPs) and megakaryocyte/erythrocyte progenitors (MEPs) increased in the *Cpt1a*^Δ*/*Δ^ BM, whereas CMPs increased in *Cpt1a*^Δ*/*Δ^ spleens (Figure 1, H and I). Furthermore, we performed a CFU assay and found decreased colony numbers after the third replating of *Cpt1a*^Δ*/*Δ^ BM cells (Figure 1, J and K).

Considered together, these data underscore the important autonomous role of *Cpt1a* in maintaining hematopoiesis.

### Cpt1a^Δ/Δ^ augments early erythroid progenitors but is dispensable in terminal erythroid differentiation.

**Figure 2:**
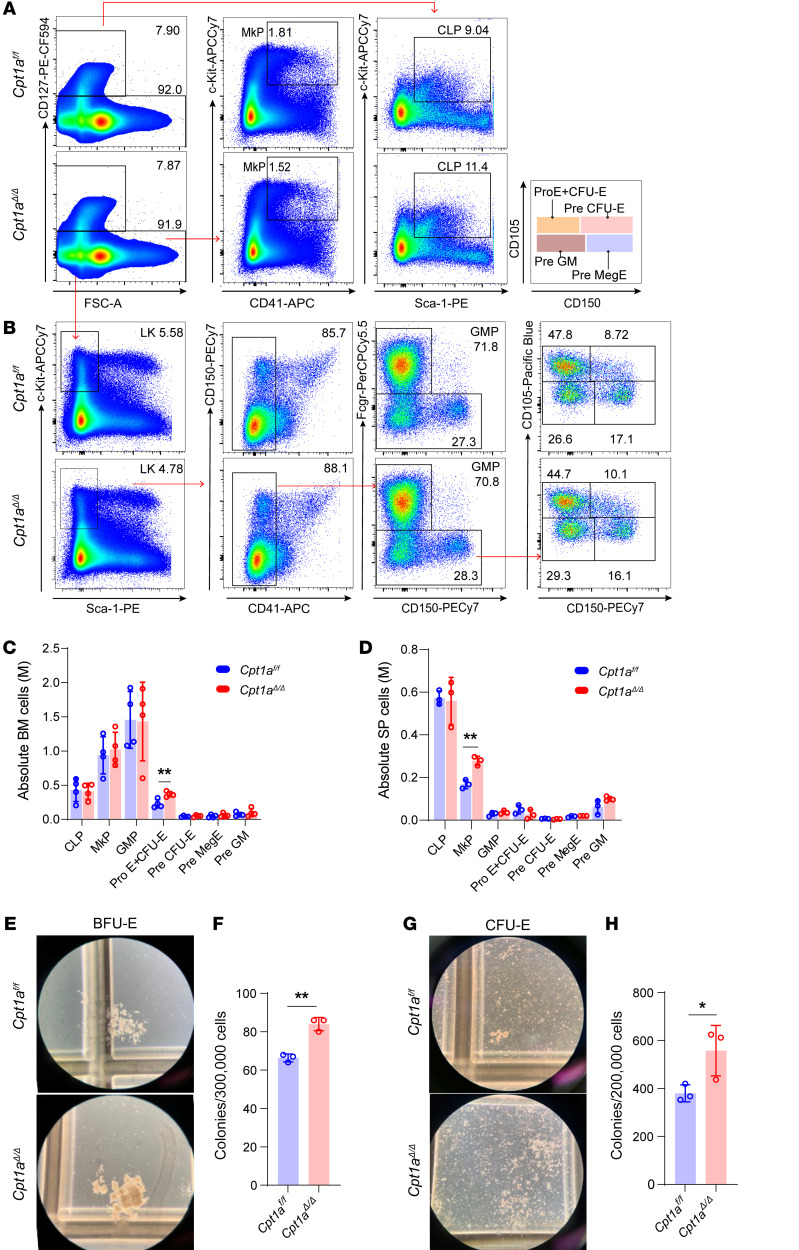
*Cpt1a^Δ/Δ^* augments early erythroid progenitors but is dispensable for terminal erythroid differentiation. (**A** and **B**) Representative flow cytometric analysis of HSPC populations in the BM. (**C** and **D**) Absolute numbers of HSPCs in the BM and spleen: MkP (Lin^–^CD127^–^c-Kit^+^CD41^+^), Pro E+CFU-E (Lin^–^CD127^–^Sca-1^–^c-Kit^+^CD41^–^Fcgr^–^CD105^+^ CD150^–^), Pre CFU-E (Lin^–^CD127^–^Sca-1^–^c-Kit^+^CD41^–^Fcgr^–^CD105^+^CD150^+^), Pre MegE (Lin^–^CD127^–^Sca-1^–^c-Kit^+^CD41^–^Fcgr^–^CD105^–^CD150^+^), Pre GM (Lin^–^CD127^–^Sca-1^–^c-Kit^+^CD41^–^Fcgr^–^CD105^–^CD150^–^). *n* = 5 per group. (**E**) BFU-E colonies. *n* = 3 per group. (**F**) Statistics of BFU-E colonies. (**G**) CFU-E colonies. Original magnification, ×100 (E and G). (**H**) Statistics for CFU-E colonies. *n* = 3 per group. Data represent the mean ± SEM. *P < 0.05 and **P < 0.01, by Student’s *t* test. Pre MegE, megakaryocyte/erythrocyte; Pre GM, pre-granulocyte/macrophage.

To understand the relationship of megakaryocytes and erythroid progenitors, respectively, to increased MEPs and decreased platelets in the BM of *Cpt1a*^Δ*/*Δ^ mice, we utilized a staining strategy to subdivide the megakaryocytes and erythroid progenitors (Figure 2, A and B). Our findings indicated elevated pro-erythroblast plus CFU-erythroid (Pro E+CFU-E) populations in *Cpt1a*^Δ*/*Δ^ BM and increased megakaryocyte progenitors (MkPs) in *Cpt1a*^Δ*/*Δ^ spleens (Figure 2, C and D). There were no noticeable differences in absolute numbers or frequencies of other major progenitor populations in the BM and spleen (Figure 2, C and D, and Supplemental Figure 5, C and D). However, burst-forming unit–erythroid (BFU-E) and CFU-E colony assays revealed increased colonies in *Cpt1a*^Δ*/*Δ^ BM cells (Figure 2, E–H).

To further understand the differentiation of progenitor cells, we analyzed terminal erythroid cell differentiation in the BM and the spleen; the various stages of nucleated erythroblasts showed no differences in the BM or spleen in *Cpt1a*^Δ*/*Δ^ mice, as compared with *Cpt1a^fl/fl^* control mice (Supplemental Figure 5, E–I). Except for erythroid cell differentiation, other mature lineage cells in peripheral blood (PB), BM, and the spleen showed no differences (Supplemental Figure 6, A–E).

Taken together, these findings suggest that loss of *Cpt1a* increased early erythroid progenitor numbers but was dispensable for the terminal differentiation of erythrocytes.

### Cpt1a^Δ/Δ^ HSCs have compromised reconstitution and self-renewal capacities.

**Figure 3:**
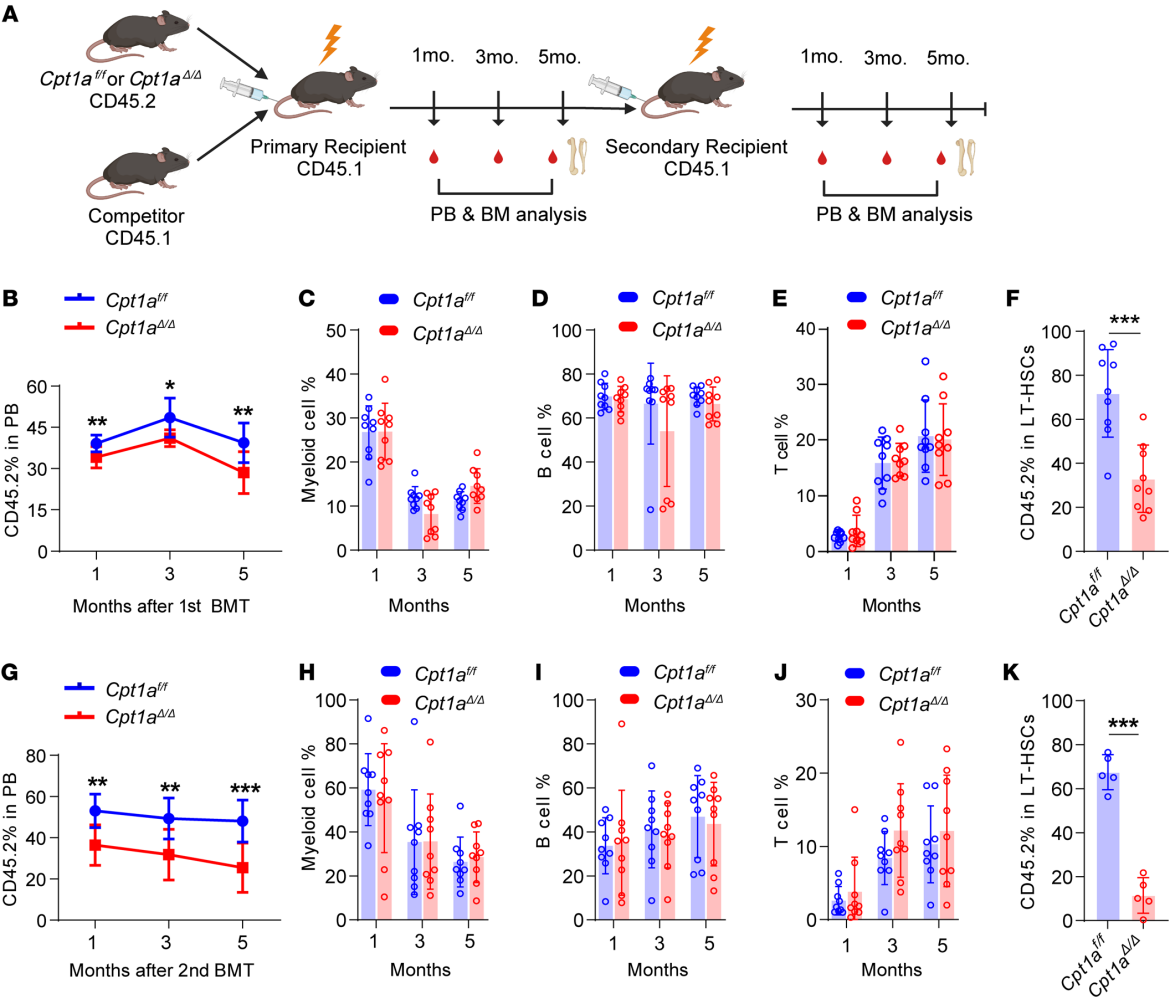
*Cpt1a^Δ/Δ^* HSCs have compromised reconstitution and self-renewal capacities. (**A**) Schematic for competitive and secondary BMTs (created with BioRender.com). (**B**) Percentage of donor-derived CD45.2 chimerism in the PB of primary recipients. *n* = 9 per group. (**C**–**E**) Chimerism of mature cells, including myeloid cells (Gr1^+^CD11b^+^), B cells (B220^+^), and T cells (CD4^+^CD8^+^) in the PB of primary recipients. *n* = 9 per group. (**F**) Frequencies of donor-derived CD45.2 LT-HSCs in CD45.2 BM, 5 months after primary BMT. *n* = 9 per group. (**G**) Percentage of donor-derived CD45.2 chimerism in PB after secondary BMT. *n* = 9 per group. (**H**–**J**) Chimerism of mature cells, as indicated, in the PB of secondary recipients. *n* = 9 per group. (**K**) Frequencies of the indicated donor-derived CD45.2 LT-HSCs in CD45.2 BM, 5 months after secondary BMT. Data represent the mean ± SEM. *P < 0.05, **P < 0.01, and ***P < 0.001, by Student’s *t* test.

To determine the effect of the *Cpt1a*^Δ*/*Δ^ conditional KO on HSC self-renewal capacity, we performed serial competitive BM transplantation (BMT) assays (Figure 3A). We monitored CD45.2 PB chimerism every 2 months beginning the first month after transplantation. CD45.2 PB chimerism of *Cpt1a*^Δ*/*Δ^ transplant recipients was markedly lower than that of *Cpt1a^fl/fl^* transplant recipients (Figure 3B). CD45.2 PB chimerism in mature lineage cells, including myeloid cells, B cells, and T cells displayed no noteworthy differences between *Cpt1a^fl/fl^* and *Cpt1a*^Δ*/*Δ^ mice (Figure 3, C–E). At the end of the initial BMT, CD45.2 BM chimerism exhibited a trend consistent with that observed in PB (Figure 3F). These results suggest that *Cpt1a*^Δ*/*Δ^ reduced the reconstitution capacity of HSCs.

Furthermore, secondary transplantations were performed 5 months after the initial BMT (Figure 3A). Lower frequencies of CD45.2 PB chimerism were consistently observed using BM from *Cpt1a*^Δ*/*Δ^ mice in initial transplants, mirroring the initial BMT results (Figure 3G). In PB, mature lineage cells remained mostly unchanged (Figure 3, H–J). There was also a lower frequency of CD45.2 chimerism for BM from *Cpt1a*^Δ*/*Δ^ mice, as compared with *Cpt1a^fl/fl^* mice (Figure 3K). In the secondary transplantation experiments, the assessment of CD45.2 chimerism in PB and BM revealed an inability of *Cpt1a*^Δ*/*Δ^ HSCs to achieve long-term reconstitution in the recipients.

Taken together, our results demonstrate that *Cpt1a* deficiency impaired the intrinsic self-renewal capacity of HSCs.

### Loss of quiescence of Cpt1a^Δ/Δ^ HSCs and sensitivity to genotoxic stress.

**Figure 4:**
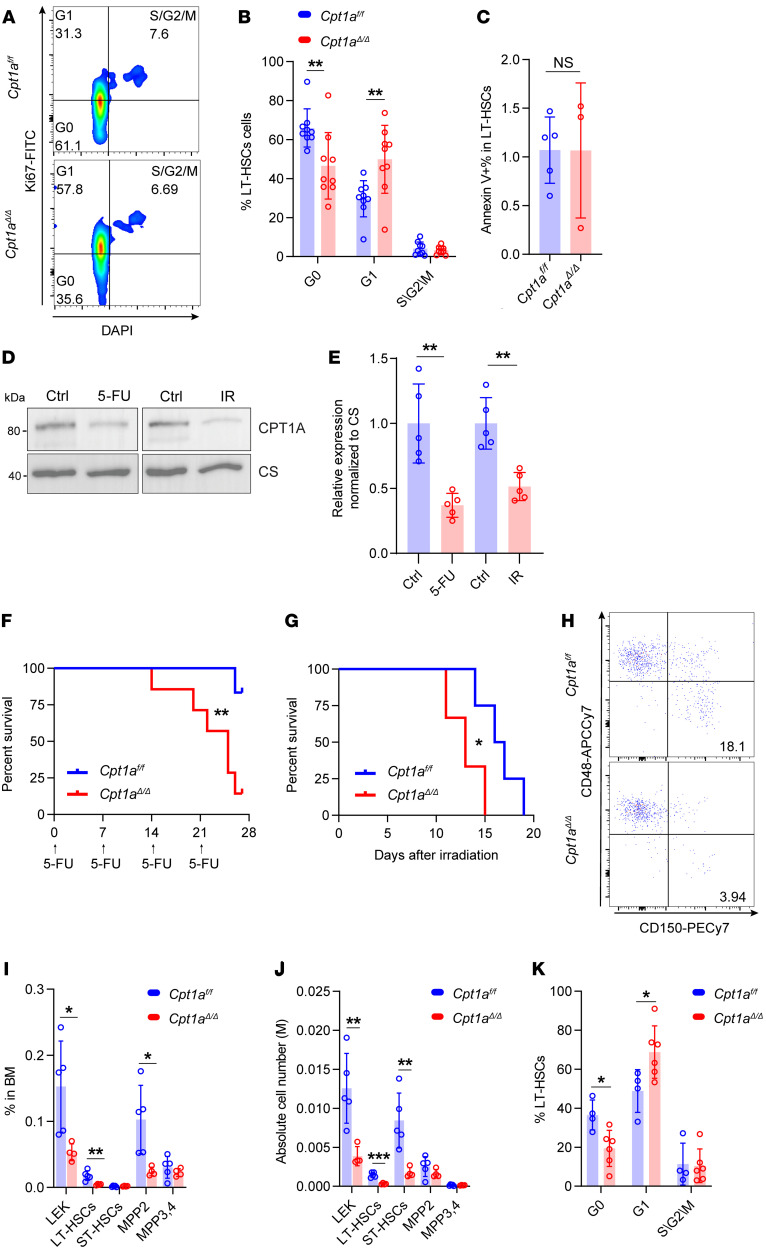
Loss of quiescence in *Cpt1a^Δ/Δ^* HSCs and hypersensitivity to genotoxic stress. (**A**) Representative flow cytometric analyses of the cell cycle following staining with Ki67 and DAPI in LT-HSCs. (**B**) Frequencies of different cell-cycle stages in LT-HSCs in the BM. *n* = 9 per group. (**C**) Apoptosis measured by annexin V expression in LT-HSCs. *n* = 5 or 3. (**D**) Western blot analysis of CPT1A expression in control (Ctrl) mice, 5-FU–treated mice, and γ-IR–treated (IR) mice, 6 days after treatment. Representative data from 5 independent experiments are shown. (**E**) CPT1A expression in control mice, 5-FU–treated mice, and γ-IR–treated mice. *n* = 5 per group. (**F**) Kaplan-Meier survival curves for mice after serial i.v. 250 mg/kg doses of 5-FU treatment at 7-day intervals. *n* = 13 per group. (**G**) Kaplan-Meier survival curves of mice after 9.5 Gy γ-IR. *n* = 7 per group. (**H**) Representative flow cytometric analysis of HSCs gated from the LEK population after 5-FU treatment. Cell populations were the same as those shown in Figure 1D. (**I** and **J**) Frequencies and absolute numbers of HSCs in BM cells 7 days after 5-FU treatment. *n* = 5 per group. (**K**) Frequencies of LT-HSCs at different cell-cycle stages, 7 days after 5-FU treatment. *n* = 4 or 6. Data represent the mean ± SEM. *P < 0.05 and **P < 0.01, by Student’s *t* test.

Given our findings that *Cpt1a* deficiency led to a diminished self-renewal capacity in HSCs (Figure 3), we sought to explore whether the quiescent state was altered. For this purpose, we analyzed the cell-cycle status of HSCs (Figure 4A). In LT-HSCs, we observed a decrease in the G_0_ phase, accompanied by an increase in the G_1_ phase in *Cpt1a*^Δ*/*Δ^ mice (Figure 4B). Furthermore, we found that the reduced numbers of LT-HSCs in *Cpt1a*^Δ*/*Δ^ mice were not attributable to heightened apoptosis, as similar rates of apoptosis were detected in LT-HSCs from *Cpt1a^fl/fl^* and *Cpt1a*^Δ*/*Δ^ mice (Figure 4C). These findings suggest that the effect of *Cpt1a*^Δ*/*Δ^ on LT-HSCs involved a transition from quiescence to proliferation, independent of apoptosis.

Following stress, HSCs are activated from quiescence, resulting in damage to their self-renewal capacity (25, 26). To elucidate the role of CPT1A in HSCs under genotoxic stresses, we assessed the expression of CPT1A in mitochondria after 5-FU treatment, a chemo regimen recognized for inducing HSC exhaustion (26), and following exposure to γ-irradiation (γ-IR). Interestingly, we found that CPT1A expression was reduced under these genotoxic stresses (Figure 4, D and E, and Supplemental Figure 7, A–D). We also compared the tolerance of *Cpt1a^fl/fl^* and *Cpt1a*^Δ*/*Δ^ mice to genotoxic stresses. After serial 5-FU treatment, *Cpt1a*^Δ*/*Δ^ mice survived only 2 cycles of half-lethal-dose injections, whereas *Cpt1a^fl/fl^* mice endured at least 4 cycles (Figure 4F). We assessed survival rates after exposure to γ-IR at a lethal dose of 9.5 Gy. The median survival duration for *Cpt1a*^Δ*/*Δ^ mice was 13 days compared with 16.5 days for *Cpt1a^fl/fl^* mice (Figure 4G).

To further investigate the differences between *Cpt1a^fl/fl^* and *Cpt1a*^Δ*/*Δ^ HSCs under genotoxic stresses, we analyzed HSC populations (Figure 4H). Notably, we observed decreases in both the frequencies (Figure 4I) and absolute numbers (Figure 4J) of LT-HSCs in *Cpt1a*^Δ*/*Δ^ mice. However, there were no differences in mature blood cell production between *Cpt1a^fl/fl^* and *Cpt1a*^Δ*/*Δ^ mice after 5-FU treatment (Supplemental Figure 7, E–J). Additionally, no differences in HSC frequencies were noted at day 12 after exposure to γ-IR (Supplemental Figure 8A).

Furthermore, there was a marked reduction in the G_0_ phase of the cell cycle, suggesting diminished quiescence within the LT-HSCs from 5-FU–treated *Cpt1a*^Δ*/*Δ^ mice (Figure 4K). The cell-cycle state had similar patterns following treatment with γ-IR, as observed with 5-FU treatment (Supplemental Figure 8B).

Collectively, these findings suggest that HSCs in *Cpt1a*^Δ*/*Δ^ mice had impaired maintenance of quiescence and increased sensitivity to a range of stressors. This strengthens the conclusion that deficiency of *Cpt1a* compromises the function of LT-HSCs.

### Transcriptional changes including an increased cell cycle and decreased FAO gene expression in Cpt1a^Δ/Δ^ HSPCs.

**Figure 5:**
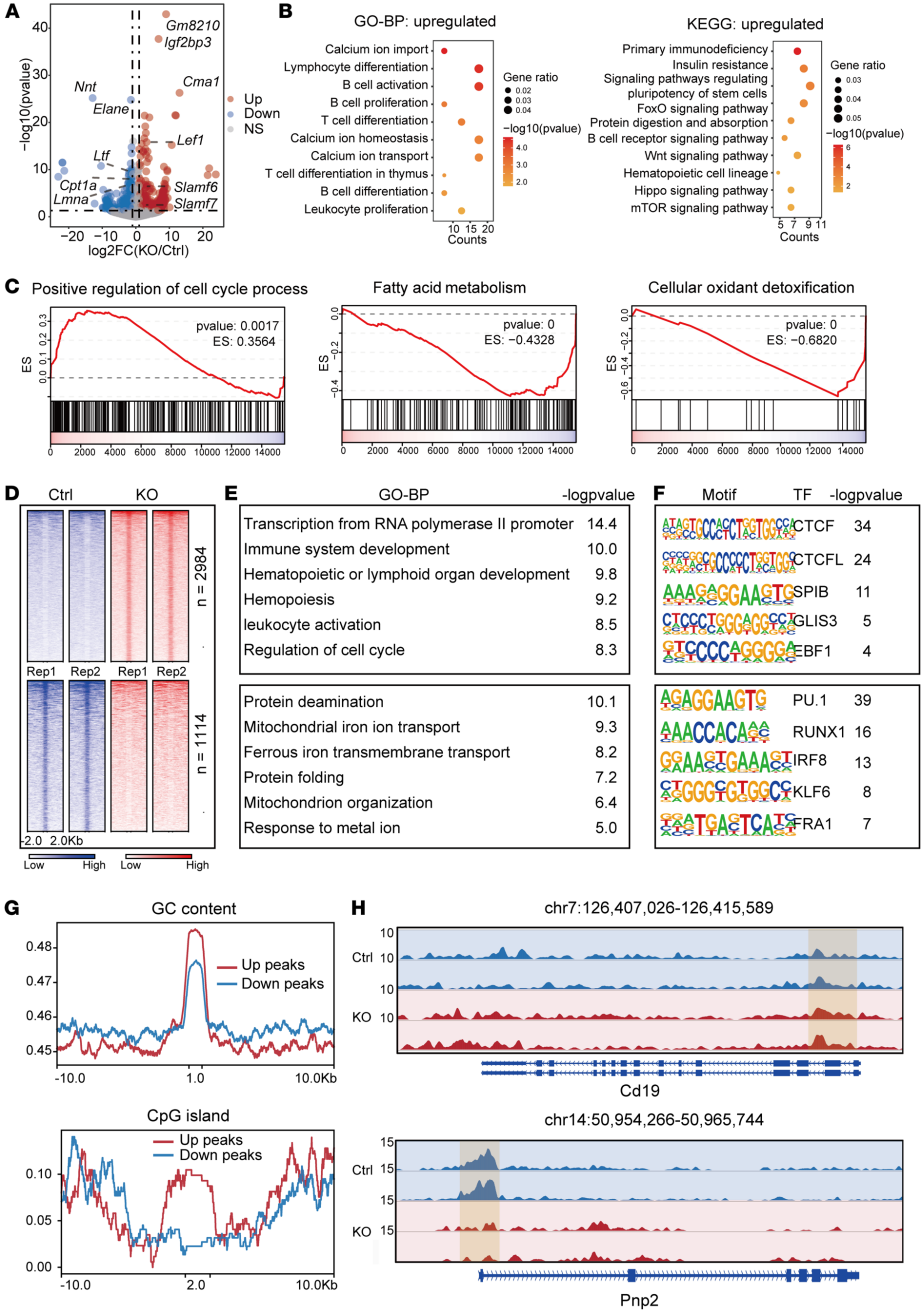
Transcriptional changes including increased expression of cell-cycle genes and decreased expression of FAO genes in *Cpt1a^Δ/Δ^* HSPCs. (**A**) Volcano plot displays differentially expressed genes (absolute log_2_ fold change >1 and *P* < 0.05) from RNA-Seq analysis. (**B**) Scatter plot shows GO-BP and KEGG pathway enrichment for upregulated genes, with the *x* axis representing the number of input genes, the size of the points indicating the gene ratio, and the color representing the enrichment level. (**C**) Representative GSEA pathways that were significantly enriched. ES, enrichment score. (**D**) Heatmap showing upregulated (up) and downregulated (down) peaks in ATAC-Seq between the control and KO groups. (**E**) GO-BP annotation of differential peaks using the GREAT web tool. (**F**) Motif analysis results for upregulated and downregulated peaks. Upregulated peaks are shown in the upper boxes, and downregulated peaks are shown in the lower boxes in **D**–**F**. TF, transcription factor. (**G**) Comparison of GC content and CpG island frequency between upregulated and downregulated peaks using metaplot. (**H**) Representative examples of differential ATAC-Seq peaks for upregulated and downregulated genes.

To elucidate the molecular mechanisms by which *Cpt1a* influences HSC function, we first performed RNA-Seq analysis of sorted *Cpt1a^fl/fl^* (control) and *Cpt1a*^Δ*/*Δ^ (KO) HSPCs. The differentially expressed genes are listed in Supplemental Table 1 (*n* = 392 downregulated and *n* = 545 upregulated; fold change >2.0). The volcano plot highlights significantly downregulated genes including *Cpt1a*, which confirmed our KO, as well as nicotinamide nucleotide transhydrogenase (*Nnt*), *Elane*, and *Lft* gene*s*, which are associated with metabolic regulation, while upregulated genes such as *Gm8210*, *Ighg3*, and *Cma1* are involved in immune functions (Figure 5A). Gene ontology biological process (GO-BP) and Kyoto Encyclopedia of Genes and Genomes (KEGG) analyses revealed enrichment of genes in calcium transport, HSC-related gene sets, metabolic signaling pathways, and immune-related processes, with these pathways predominantly involving upregulated genes (Figure 5B and Supplemental Table 2). Gene set enrichment analysis (GSEA) further confirmed that *Cpt1a*^Δ*/*Δ^ HSPCs enhanced cell-cycle regulation and cell proliferation, while suppressing pathways of fatty acid metabolism, adipogenesis, cholesterol metabolism, bile metabolism, response to ionizing radiation, and cellular oxidant detoxification (Figure 5C and Supplemental Figure 9, A–I). Overall, these findings suggest that *Cpt1a* deficiency promotes cell proliferation but compromises antioxidant defense mechanisms.

To systematically evaluate the regulatory effects of KO of the *Cpt1a* metabolic enzyme on chromatin accessibility, we conducted an assay for transposase-accessible chromatin using sequencing (ATAC-Seq) with the same sorted *Cpt1a^fl/fl^* (control) and *Cpt1a*^Δ*/*Δ^ (KO) HSPCs. We found increased chromatin accessibility in immune- and cell-cycle-related genes (up peaks, *n* = 2,984) and decreased accessibility in metabolic regions (down peaks, *n* = 1,114) (Figure 5, D and E). Motif analysis identified CCCTC-binding factor (CTCF, a chromatin organization protein) and PU.1 (also known as SPI1, a hematopoietic transcription factor) binding sites as the most enriched in upregulated and downregulated peaks, respectively, which is indicative of their roles in chromatin remodeling and subsequent transcriptional changes (Figure 5F and Supplemental Table 3). Guanine-cytosine content (GC) content and cytosine-phosphate-guanine (CpG) island analyses showed that up peaks were highly GC rich, whereas down peaks were CpG poor, indicating a selective effect of *Cpt1a*^Δ*/*Δ^ on specific genomic regions with high GC content and CpG islands (Figure 5G). Consistent with our RNA-Seq analyses, genome tracts revealed increased accessibility in differentiation-, immune-, and cell-cycle-related genes (*Cd19*) and decreased accessibility in fatty acid metabolism genes (*Pnp2*) (Figure 5H).

In summary, KO of *Cpt1a* remodeled chromatin accessibility and gene expression, which may promote immune activation and cell proliferation and differentiation but impair fatty acid metabolism and antioxidant defenses. Taken together, these findings reveal key roles for *Cpt1a* in HSCs through regulation of metabolic homeostasis, as well as chromatin accessibility and gene expression.

### Increased glucose-fueled mitochondrial function in Cpt1a^Δ/Δ^ HSCs.

**Figure 6:**
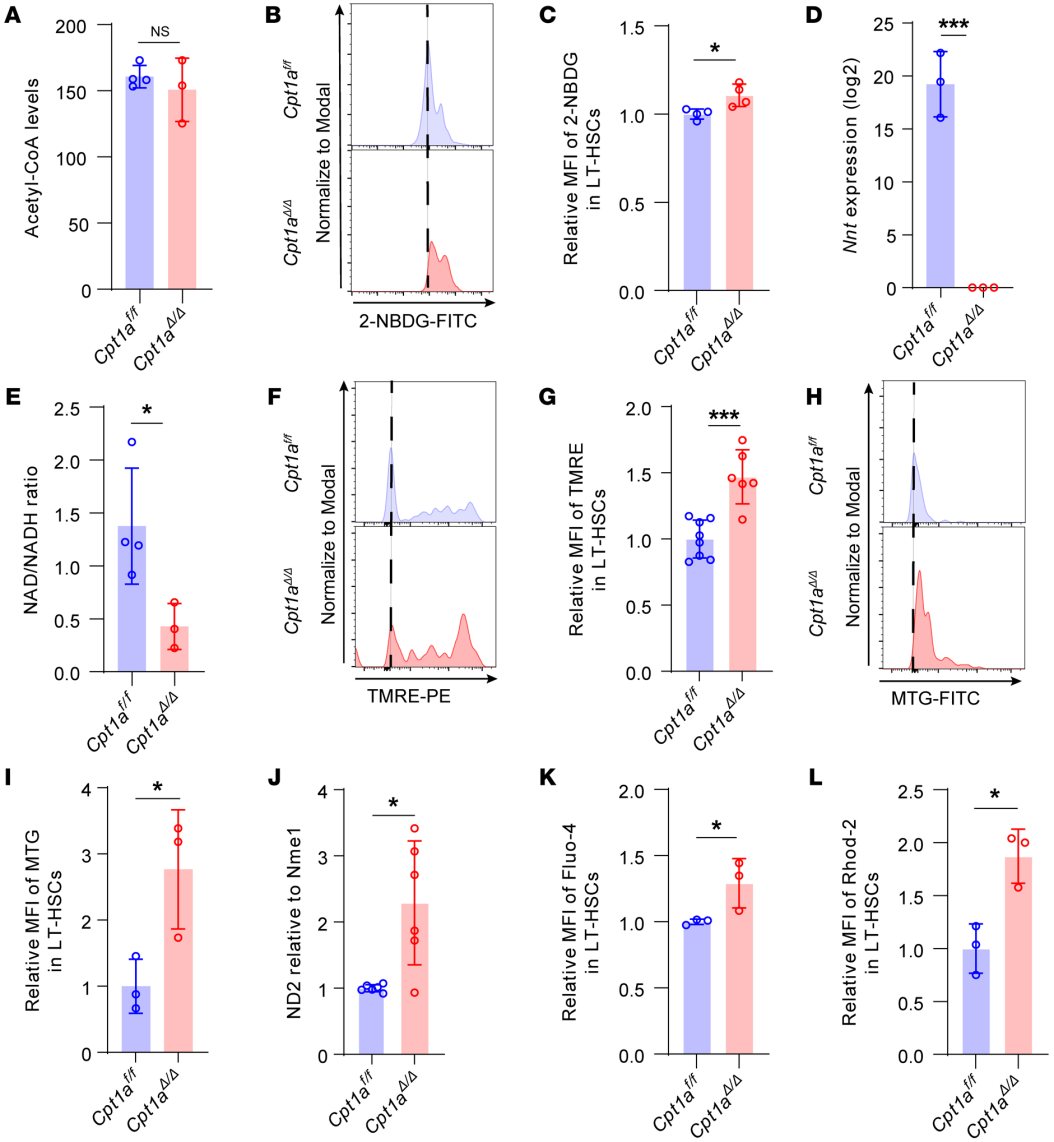
Increased glucose-fueled mitochondrial activity in *Cpt1a^Δ/Δ^* HSCs. (**A**) Acetyl-CoA levels in c-Kit^+^ cells. *n* = 4 or 3. (**B**) Representative flow cytometric analysis of glucose uptake measured via 2-NBDG staining in LT-HSCs. (**C**) Relative MFI levels of 2-NBDG signal (MFI). *n* = 4 per group. (**D**) *Nnt* expression based on RNA-Seq. *n* = 3 per group. (**E**) NAD/NADH ratio in c-Kit^+^ cells. *n* = 4 or 3. (**F**) Representative flow cytometric analysis of MMP by TMRE in LT-HSCs. (**G**) Relative MFI levels of TMRE signal. *n* = 8 or 6. (**H**) Representative flow cytometric analysis of MTG in LT-HSCs. (**I**) Relative mitochondrial mass levels were determined by MTG staining. *n* = 3 or 4. (**J**) Relative mtDNA levels (ND2) compared with nuclear DNA (Nme1) by quantitative PCR (qPCR) in purified c-Kit^+^ cells. *n* = 5 or 6. (**K**) Relative MFI levels of intracellular Ca^2+^ measured by Fluo-4, a measurement of cytosolic calcium, in LT-HSCs. n = 3 per group. (**L**) Relative MFI levels of mitochondrial Ca^2+^ measured by Rhod-2, a measurement of mitochondria-specific calcium detection, in LT-HSCs. *n* = 3 per group. Data represent the mean ± SEM. *P < 0.05 and ***P < 0.001, by Student’s *t* test.

CPT1A regulates the entry of fatty acids into mitochondria for β-oxidation, facilitating their metabolism into acetyl-CoA, which can then enter the TCA cycle to couple with mitochondrial OXPHOS for ATP and NADPH production (18). To elucidate the effects of *Cpt1a* deficiency and compromised fatty acid intake on the metabolic state of HSCs, we measured acetyl-CoA levels and found that these levels were slightly decreased in *Cpt1a*^Δ*/*Δ^ HSCs but not statistically significant (Figure 6A).

Next, we measured glucose uptake to assess glycolytic activity using the glucose analog 2-(*N*-(7-nitrobenz-2-oxa-1,3-diazol-4-yl)amino)-2-deoxyglucose (2-NBDG) (Figure 6B). We observed that 2-NBDG uptake was significantly increased in *Cpt1a*^Δ*/*Δ^ mice compared with *Cpt1a^fl/fl^* mice (Figure 6C), with enhancements also noted in *Cpt1a*^Δ*/*Δ^ mice in response to genotoxic stress (Supplemental Figure 8, C and D).

RNA-Seq analysis revealed that *Nnt* expression was significantly reduced in *Cpt1a*^Δ*/*Δ^ HSPCs (Figure 6D). NNT is an enzyme that transfers reducing equivalents from NADH to NADPH (27). To further investigate the metabolic implications of reduced *Nnt* expression, we measured the NAD/NADH ratio and observed a decrease in *Cpt1a*^Δ*/*Δ^ mice, consistent with downregulation of *Nnt* (Figure 6E). We speculate that this suppression may be allosterically mediated by elevated levels of NADH (27). Taken together, these results show maintenance of acetyl-CoA levels and increased glucose intake, indicating that enhanced glycolysis may compensate for FAO deficiency in mitochondrial metabolism and energy production.

We further evaluated changes in mitochondrial function by mitochondrial membrane potential (ΔΨm), which is essential to energy storage and ATP production during OXPHOS (28), using the tetramethylrhodamine ethyl ester (TMRE) probe in LT-HSCs (Figure 6F). Increases in glucose uptake and TMRE were also observed in mice under genotoxic stress (Supplemental Figure 8, E and F). Interestingly, this analysis revealed markedly elevated ΔΨm levels in LT-HSCs from *Cpt1a*^Δ*/*Δ^ mice (Figure 6G). To analyze whether mitochondrial biogenesis was responsible for the increase in mitochondrial function, we utilized the MitoTracker Green (MTG) staining assay, which revealed an increase in mitochondrial mass in *Cpt1a*^Δ*/*Δ^ mice (Figure 6, H and I). This finding was also corroborated by quantifying the mitochondrial DNA (mtDNA) copy number, as described previously (29, 30). Specifically, we found that the ratio of the mtDNA, NADH dehydrogenase subunit 2 (ND2), to the nuclear DNA, NME/NM23 nucleoside diphosphate kinase 1 (Nme1), was elevated in c-Kit^+^ cells from *Cpt1a*^Δ*/*Δ^ mice (Figure 6J). Previous studies indicated that increased intracellular calcium (Ca^2+^) enhanced mitochondrial activity in cycling HSCs (22, 31). We examined cellular versus mitochondrial Ca^2+^ flux using Fluo-4 and Rhod-2 staining, respectively. We observed an increase in both cellular and mitochondrial Ca^2+^ flux in LT-HSCs in *Cpt1a*^Δ*/*Δ^ mice (Figure 6, K and L).

Taken together, these data suggest that mitochondrial activity, including glucose uptake, mitochondrial membrane potential, mitochondrial mass, and Ca^2+^ concentration, was increased in *Cpt1a*^Δ*/*Δ^ HSCs and was accompanied by an altered metabolic state.

### Increased levels and activity of respiratory complex components in Cpt1a^Δ/Δ^ HSCs.

**Figure 7:**
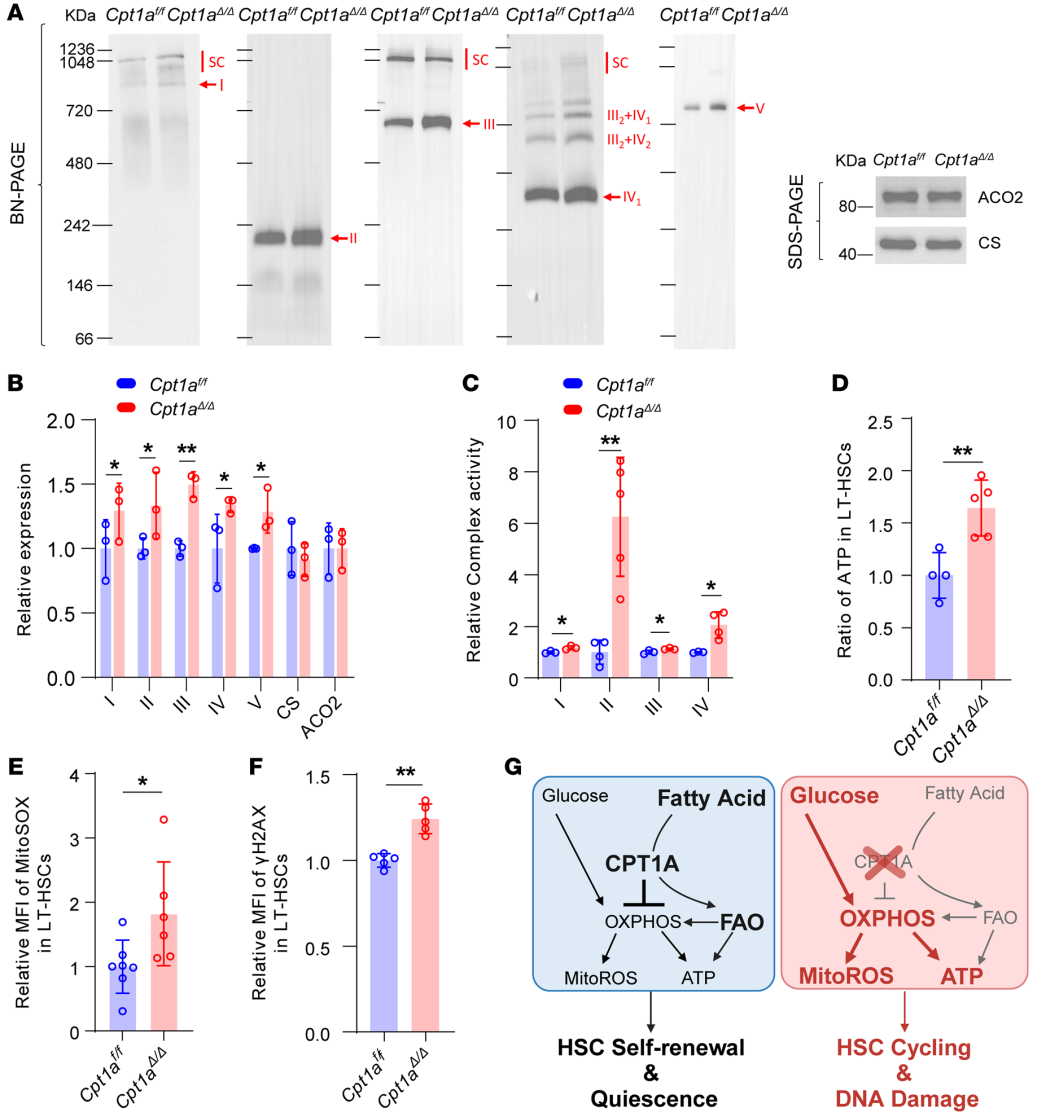
Increased levels and activity of mitochondrial respiratory complex components in *Cpt1a^Δ/Δ^* HSCs. (**A**) Left: BN-PAGE immunoblots of mitochondrial complexes and supercomplexes contents in mitochondria of c-Kit^+^ cells. The supercomplexes were visualized by antibodies against subunits of complex I (NDUFA9), complex II (SDHA), complex III (UQCRC2), complex IV (COX4a), and complex V (ATP5A1). Representative data from 3 independent experiments are shown. Right: SDS-PAGE immunoblots of the mitochondrial internal controls mitochondrial ACO2 and CS in c-Kit*^+^* cells. Representative data from 3 independent experiments are shown. (**B**) Relative expression of complexes I–V, CS, and ACO2 in c-Kit^+^ cells. *n* = 3 per group. (**C**) Relative activity of complexes I, II, III, and IV was measured by ELISA in c-Kit^+^ cells. *n* = 4 or 5. (**D**) Relative ATP levels detected by ELISA of LT-HSCs. *n* = 4 or 5. (**E**) Relative MFI levels of mtROS measured by MitoSOX in LT-HSCs. *n* = 7 or 6. (**F**) Relative MFI levels of γH2A.X in LT-HSCs. *n* = 5 per group. (**G**) Diagrammatic summary of metabolic reprogramming in HSCs (created with BioRender.com). Data represent the mean ± SEM. *P < 0.05 and **P < 0.01, by Student’s *t* test.

ΔΨm, mtROS, and ATP production are all related to OXPHOS and the electron transport chain (ETC), which is also known as the respiratory chain (32). To further investigate the mechanism underlying the increase in OXPHOS products in *Cpt1a*^Δ*/*Δ^ HSCs, we analyzed the levels and activity of the various ETC complexes. We first isolated mitochondria from HSCs and quantified the ETC complexes using a blue native PAGE (BN-PAGE) assay, ensuring that equal amounts of mitochondria were used for each group (33, 34). We observed increased levels of each of complexes I, II, III, IV, and V in *Cpt1a*^Δ*/*Δ^ HSCs compared with *Cpt1a^fl/fl^* controls, using mitochondrial aconitase (ACO2) and citrate synthase (CS) as internal controls (Figure 7, A and B).

Next, we demonstrated that *Cpt1a*^Δ*/*Δ^ HSCs had enhanced activity of complexes I, II, III, and IV, with a particularly notable 6-fold increase in complex II (succinate dehydrogenase [SDH]) activity (Figure 7C). Additionally, we observed an increase in intracellular ATP content in *Cpt1a*^Δ*/*Δ^ LT-HSCs (Figure 7D).

We also measured mtROS via MitoSOX staining, which showed a pronounced increase in *Cpt1a*^Δ*/*Δ^ LT-HSCs (Figure 7E), consistent with observations made under genotoxic stress (Supplemental Figure 8, G and H). However, we observed no differences when we assessed total cellular ROS using the CellROX assay (Supplemental Figure 8I). Furthermore, we investigated whether the increase in mtROS correlates with the accumulation of DNA damage. Our observations reveal that DNA damage–induced γH2A.X levels were significantly elevated in *Cpt1a*^Δ*/*Δ^ LT-HSCs (Figure 7F). Thus, we identified metabolic reprogramming in *Cpt1a*^Δ*/*Δ^ HSCs that was characterized by elevated ATP production and increased mtROS generation, driven by enhanced mitochondrial respiration fueled by glucose (Figure 7G).

Collectively, our results suggest that CPT1A restrained OXPHOS, potentially by promoting FAO-based energy production.

## Discussion

This study reveals a critical role for CPT1A in the regulation of mitochondrial metabolic function in HSCs and, in turn, the control of HSC function. For example, our observations suggest that the *Cpt1a*^Δ*/*Δ^ led to a loss of quiescence and a reduction in LT-HSC populations and, ultimately, to an increase in HPC differentiation in *Cpt1a*^Δ*/*Δ^ mice. These effects are primarily attributable to mitochondrial reprogramming, resulting in increased mitochondrial biogenesis and OXPHOS activity.

Despite extensive research on the role of CPT1A in fatty acid metabolism, its specific function in HSCs remains poorly understood. Our observations reveal that CPT1A was highly expressed in LT-HSCs as compared with BM and lineage^+^ (Lin^+^) cells, suggesting the possibility of a distinct role in HSC biology. Consistent with our findings, a previous study reported *Cpt1a* upregulation in the LSK population at the mRNA level (35).

HSCs can differentiate to form various blood cell types (36). *Cpt1a*-dependent FAO is required for neurogenesis (12). Our study showed that mature cell lineages were mostly unaffected by *Cpt1a* deletion, and it has been reported that a specific deficiency in T cells does not affect T cell homeostasis or activation (24). Nevertheless, our results show that early erythroid progenitors were increased in *Cpt1a*^Δ*/*Δ^ mice However, despite lipids being used as fuels by erythroblasts at erythropoiesis (37), we observed negligible changes in late erythroid progenitors and mature erythroid cells in *Cpt1a*^Δ*/*Δ^ mice. All the same, the mechanism through which *Cpt1a* influences early erythropoiesis warrants further exploration.

*Cpt1a*^Δ*/*Δ^ mice exhibited compromised reconstitution with HSCs following BMT and increased sensitivity to stress, further evidenced by the diminished presence of LT-HSCs in the quiescent G_0_ phase. Thus, these results demonstrate a previously unrecognized function for CPT1A and FAO in regulating HSC quiescence and self-renewal.

*Cpt1a* has been reported to be associated with genome-wide methylation in acute myeloid leukemia, alterations in acetyl-CoA levels, and H3K27ac at open chromatin sites in embryonic stem cells (38, 39). Our current study found that *Cpt1a* deficiency in HSPCs reshaped chromatin accessibility and changed gene expression, enhancing cell proliferation while impairing fatty acid metabolism and oxidative defenses, thus highlighting its essential role in maintaining HSC function and metabolic balance.

Dysfunction of mitochondrial metabolism has been implicated in the disruption of HSC maintenance (16, 17, 40). NNT is essential for maintaining mitochondrial function and redox balance (41). Knockdown of NNT reduces glutamine contribution to the TCA cycle, while promoting glucose metabolism via the TCA cycle through an altered NAD(P)H/NAD(P) balance (27). Consistent with these possibilities, our study demonstrated an elevation in glucose uptake, ATP levels, and ΔΨm and mtROS levels, **along** with a decrease in NNT expression and the NAD/NADH ratio in *Cpt1a*^Δ*/*Δ^ HSCs. All these events related to energy metabolism were upregulated in *Cpt1a*^Δ*/*Δ^ LT-HSCs, which indicates reprogramming of energy production in these cells. The enhancement of mitochondrial OXPHOS in the absence of FAO may be due to energy demand and an imbalance in the total ATP pool. It has been reported that elevated ΔΨm and ATP, which are regulated by enhanced Ca^2+^ levels, could drive HSC division (22, 31). Correspondingly, we found that cellular and mitochondrial Ca^2+^ levels were augmented and that the Ca^2+^ import pathway was upregulated in RNA-Seq data as well as in *Cpt1a*^Δ*/*Δ^ LT-HSCs.

Mitochondrial membrane potential (ΔΨm) serves as a critical indicator of mitochondrial function, primarily driven by redox reactions facilitated by ETC complexes I, III and IV, and ultimately leading to ATP synthesis via ATP synthase (complex V) (19, 28). Elevated ΔΨm and ATP levels imply enhanced OXPHOS activity. NADH is a common reducing equivalent for both NNT and complex I, and an increased NADH/NAD ratio leads to complex I dysfunction and increased ROS production (41, 42). Increased levels of mtROS, a byproduct of OXPHOS, further corroborated OXPHOS activation in *Cpt1a*^Δ*/*Δ^ HSCs. This increase was also accompanied by downregulation of the pathway that detoxified ROS. Consistent with these observations, in our study, *Cpt1a*^Δ*/*Δ^ HSCs exhibited heightened levels and activity of various ETC complexes, signifying a metabolic shift from FAO toward glycolysis and glucose-fueled OXPHOS. This mitochondrial metabolic reprogramming in *Cpt1a*^Δ*/*Δ^ HSCs appeared to compromise HSC maintenance, underscoring the importance of the balance between FAO and glucose-fueled OXPHOS in stem cell homeostasis.

In conclusion, our findings illuminate the intricate connections among *Cpt1a*, mitochondria, and LT-HSCs. Furthermore, *Cpt1a* emerged as a pivotal player in the maintenance of HSC homeostasis by modulating mitochondrial function. Despite our findings that various mitochondrial properties were altered in LT-HSCs, continued investigation will be required to unravel the detailed mechanisms and precise relationship between *Cpt1a* and mitochondrial energy production in HSCs. Finally, our results suggest the possibility that *Cpt1a* activity may serve as a potential target for addressing HSC exhaustion under stresses and may offer an essential means to sustain functional HSCs.

## Methods

### Sex as a biological variable.

Adult mice (8–12 weeks old) of both sexes were used unless otherwise stated. Sex was not considered as a biological variable in this study.

### Animals.

*Cpt1a*-floxed (*Cpt1a^fl/fl^*) mice (RRID: IMSR_JAX:032778) and *Vav1-Cre^+^* mice were purchased from The Jackson Laboratory. *Cpt1a*^Δ*/*Δ^ mice were generated by crossing *Cpt1a^fl/fl^* mice with *Vav1-Cre^+^* mice, and all were maintained on a C57BL/6, CD45.2 background. C57BL/6, CD45.1 mice that served as recipients in BMT assays were obtained from the CCHMC/Cancer and Blood Diseases Institute mouse core. All mice were housed in the rodent barrier facility at CCHMC and UT Health San Antonio.

### Flow cytometry.

Whole BM cells were flushed from the bones (tibia, femur, and/or pelvis) with Hanks’ buffer (Gibco, Thermo Fisher Scientific, 14170112). Single splenic cell suspensions were isolated from crushed whole spleens using a 40 μM strainer, and PB was collected by retro-orbital puncture. Unless otherwise stated, single-cell suspensions were prepared using FACS buffer (DPBS (Gibco, Thermo Fisher Scientific, 14190094) with 2% heat-inactivated bovine serum (Atlanta, S11550H), followed by RBC lysis (BD Biosciences, 555899). Analysis was performed on a BD Fortessa I flow cytometer, and the results were analyzed using FlowJo, version 10, software. Staining strategies and materials can be found in the Supplemental Methods.

### BMT assays.

Six- to 8-week-old CD45.1 mice were lethally γ-irradiated (9.5 Gy). For competitive transplantation, 1 million CD45.1 competitor BM cells were mixed with 1 million CD45.2 *Cpt1a^fl/fl^* or *Cpt1a*^Δ*/*Δ^ BM cells. Mice were transplanted with BM cells via tail vein injection within 24 hours of γ-IR. Chimerism and lineage contribution in PB were analyzed by flow cytometry at 1, 3, and 5 months after transplantation. In the fifth month after transplantation, the chimerism of HSCs was analyzed by flow cytometry. For secondary transplantation, 1 million BM cells were transplanted into secondary recipient mice. Chimerism and lineage contribution in PB and HSCs were performed the same way as for the first transplantation.

### RNA-Seq and ATAC-Seq.

RNA-Seq was performed using the HiSeq 4000 platform (Illumina). Differentially expressed genes were identified using DESeq2 (43), with genes exhibiting an absolute log_2_ fold change of greater than 1 and a *P* value of less than 0.05 considered significant. Differentially expressed genes were subjected to enrichment analysis for GO biological processes and KEGG pathways using the clusterProfiler package (44). Differential peaks were identified using the DiffBind package (45) and analyzed for statistical significance with edgeR (46), applying a FDR of less than 0.01 and an absolute log_2_ fold change of greater than 1. Upregulated and downregulated peaks were annotated for GO biological processes using the Genomic Regions Enrichment of Annotations Tool (GREAT) web tool (47). Hypergeometric Optimization of Motif EnRichment (HOMER) was used for motif analysis (48). The genomic GC content and CpG island proportions were calculated using Bedtools (49). DeepTools (50) was used to generate bigWig files, which were then visualized as heatmaps. The resulting data tracks were visualized using IGV Tools.

### Glucose uptake, mitochondrial superoxide, and mitochondrial mass.

HSCs were first stained with specific markers. Glucose uptake was assessed using the 2-NBDG Glucose Uptake Assay kit (BioVision, K682). Mitochondrial superoxide was quantified using MitoSOX Red Mitochondrial Superoxide Indicator (Thermo Fisher Scientific, M36008), and mitochondrial mass was determined with MitoTracker Green (Thermo Fisher Scientific, M7514). Cells were stained with 2 μM TMRE, 5 μM MitoSOX Red, and 25 nM MTG, depending on the assay, at 37°C for 30 minutes and then acquired on a Fortessa I instrument within 60 minutes of mitochondrial staining.

### Analysis of mitochondrial complexes and supercomplexes.

Mitochondrial complexes and supercomplexes were analyzed according to previously published protocols (34, 51, 52). Protein (20 μg/well) was loaded into 10-well 3%–12% BN-PAGE gels (Thermo Fisher Scientific, BN1001BOX) in a Mini Gel Tank (Thermo Fisher Scientific, A25977) and then run and transferred. Anti-GRIM19 (Abcam ab110240, 1:500), anti-SDHA (Abcam, ab14715, 1:2,000), anti-UQCRC1 (BioLegend, 696202, 1:1,000), anti-MTCO1 (Abcam, ab14705, 1:1,000), and anti-ATP5A (Abcam, ab14748, 1:1,000) antibodies were used to detect mitochondrial respiratory chain complexes I, II, III, IV, and V separately.

### Complex I, complex II, complex III, and complex IV activity.

Measurements of complex I activity (Complex I Enzyme Activity Microplate Assay Kit, ab109721, Abcam), complex II activity (Complex II Enzyme Activity Microplate Assay Kit, ab109908, Abcam), complex III activity (Mitochondrial Complex III Activity Assay Kit, ab287844, Abcam), and complex IV activity (Complex IV Rodent Enzyme Activity Microplate Assay Kit, ab109911, Abcam) in c-Kit^+^ cells were done according to the manufacturer’s instructions. Activity is expressed as the change in absorbance per minute (mOD/min) per 200 μg cell lysate.

### Statistics.

The experimental data were analyzed with GraphPad Prism 10 (GraphPad Software). Unless otherwise stated, results were obtained from at least 3 independent experiments. An unpaired, 2-tailed Student’s *t* test was used for comparisons between two groups. A log-rank nonparametric test determined the Kaplan-Meier survival curves. All data are presented as the mean **±** SD or the median (range). *P* values of less than 0.05 were considered statistically significant.

### Study approval.

All animal care and experimental procedures complied with the animal care guidelines approved by the IACUC of CCHMC (IACU2021-0019) and UT Health San Antonio (TR202400000003).

### Data availability.

RNA-Seq and gene expression data for all the samples have been deposited in the NCBI Gene Expression Omnibus (GEO) database (GEO GSE282231). ATAC-Seq and peak calling results for all the samples have been deposited in the GEO database (GEO GSE282229). The values underlying the data presented in each graph are included in the Supporting Data Values file.

## Author contributions

JL and GH conceived the project. JL, SJL, YL, HZ, PRA, and GH designed experiments, interpreted results, and revised the manuscript. JL performed experiments, analyzed data, and wrote the manuscript. JB, VTP, MH, MS, AA, TL, and MP contributed to the experiments. YL, CHH, QSJ, and QLS performed the ATAC-Seq library preparation and conducted downstream analyses of ATAC-Seq and RNA-Seq data. All authors reviewed and approved the manuscript.

## Supplementary Material

Supplemental data

Unedited blot and gel images

Supplemental table 1

Supplemental table 2

Supplemental table 3

Supplemental table 4

Supporting data values

## Version 1 01/09/2025

In-Press Preview

## Version 2 03/03/2025

Electronic publication
