# Supplementary material for: Loss of Cpt1a results in elevated glucose-fueled mitochondrial oxidative phosphorylation and defective hematopoietic stem cells
Source: J Clin Invest. 2025 Jan 9;135(5):e184069. doi: 10.1172/JCI184069 (PMC11870731; doi:10.1172/JCI184069)
Supplement: Supplemental data [file jci-135-184069-s217.pdf]

Supplementary

Methods and Materials

Genotyping

200 μL 50 mM NaOH was added into a 1.5 mL Eppendorf tube containing 1-2 mm portion of ear tissue or at least 1 million cells from each mouse. The samples were heated to 100 °C for 50 mins. 50 μL of 1 M Tris-Hcl (pH=6.8) was added to neutralize the reaction. The samples were vortexed thoroughly, and 1 μL of each sample was used for each genotyping PCR reaction. SapphireAmp Fast PCR Master Mix (Takara, RR350A) was employed according to the manufacturer's instructions. Primers: *Cpt1a* Forward (5'-3' CCA GGA TCC CTT TGA GCA G), *Cpt1a* Reverse (5'-3' AGG CCC AAG GAA GAA TCA AG).

Flow cytometry

Cell surface markers were stained following standard protocols.

Dead cells were excluded using Zombie Aqua (Biolegend, 423101) or PI (Biolegend, 421301). Fixation was performed with BD Fixation/Permeabilization Kit (BD Biosciences, 554714).

Hematopoietic populations were analyzed or sorted using the following antibodies. For mature lineage cell analysis, cells were incubated with biotin-labeled lineage antibody cocktails (anti-Gr-1, anti-CD11b, anti-CD4, anti-CD8, anti-Ter119, and B220), followed by staining for streptavidin, anti-c-Kit, anti-EPCR, anti-CD48, and anti-CD150 staining.

For progenitor cell analysis in BM and spleen, mature lineage cells were stained as described, followed by staining with streptavidin, anti-CD127, anti-CD34, anti-CD41, anti-c-Kit, anti-FcγR, anti-Sca-1, anti-CD105, and anti-CD150.

For multiple lineage cell analysis, cells were incubated with antibodies (anti-CD11b, anti-CD3, anti-CD115, anti-CD135, and B220).

For CPT1A expression staining, HSCs were first stained for, then fixed and stained with anti-CPT1A (Proteintech, 15184-1-AP, 1:1000) and a secondary anti-rabbit antibody (Jackson immune research lab, 711-545-152, 1:1000) for 30 minutes.

For cell cycle analysis with Ki67, cells were first stained for LEK-SLAM cell surface markers, fixed, and stained with Ki67 (1:100), followed by DAPI (1:1000) 10 minutes before analysis.

For BMT lineages, cells were stained with anti-CD45.1, anti-CD45.2, anti-Gr-1, anti-CD11b, anti-CD4, anti-CD8, and anti-B220.

For erythroid cells, Fc receptors were blocked for 10 minutes, and cells were incubated with biotin-labeled lineage antibody cocktails (anti-CD45, anti-Gr-1, and anti-CD11b), followed by streptavidin, anti-CD71, anti-Ter119, and anti- CD44 staining. Details of antibodies were listed in Supplementary Table 4.

Cell sorting

For LT-HSC isolation, BM cells were enriched for c-Kit$^+$ cells using c-Kit microbeads

(Miltenyi biotec, 130-091-224) and separated with the Automacs pro cell separator machine (Miltenyi biotec). The c-Kit$^+$ cells were stained for hematopoietic stem cells surface markers and sorted using the MA900 sorter (Sony).

Colony formation assays

For CFU assays, 2x10^4 BM cells were plated after RBC lysis and serially plated every seven days in triplicate into 1 mL MethoCult GF M3434 medium (Stem Cell Technologies, 03434). For BFU-E assays, 3x10^5 whole bone marrow cells were plated in triplicate into 1mL MethoCult GF M3436 medium (Stem Cell Technologies, 03436), with colonies scored after seven days. For CFU-E assays, 2x10^5 whole bone marrow cells were plated in triplicate into 1 mL MethoCult GF M3334 medium (Stem Cell Technologies, 03334), and colonies were scored after fourteen days. Colonies were observed with a Nikon microscope.

Mitochondria extraction

Buffer M was prepared with 19.1g mannitol, 11.9 g sucrose, 5 mL 1 M Tris/HCl (PH7.4), 1 mL 0.5 M EDTA (PH8.0) in ddH2O to a final volume of 500 mL. Buffer M1included 5 mL 5% BSA-FF, 50 µL 20% Digitonin, and 500 µL Protease Inhibitor (100x) in Buffer M to a final volume of 50 mL. The mitochondrial solubilization buffer (SB) was composed of 50 mM NaCl, 50 mM BisTris/HCl (PH7.0), 2 mM 6-aminohexanoic acid, 1mM EDTA, adjusted to pH 7.4.

Cells were transferred to a 2mL Dounce homogenizer in 1 mL buffer M1 and homogenized for 20 strokes by tight pestle. After incubating on ice for 10 minutes to solubilize the plasma membrane, the homogenate was centrifuged at 16,000 g for 10 minutes to obtain a pellet containing nuclei and mitochondria. The supernatant was removed, the pellet was weighed to determine the wet weight. The pellet was then resuspended with 1.7x v/v SB buffer, fully resuspended with a hand-held tissue homogenizer on ice, and ½ v/v of 20% Digitonin was added. The solution was solubilized for 10 min on ice, centrifuge at 22,000 g for 50 minutes at 4ºC, and the supernatant was carefully transferred to a new microcentrifuge tube. Protein concentration was determined using the Pierce BCA protein assay kit, and samples were prepared for BN-PAGE by mixing with 1/5 volume of protein loading buffer.

RT-qPCR and mitochondrial DNA

Total RNA from c-Kit$^+$ cells was isolated using the RNeasy Mini Kit (Qiagen, Hilden, Germany), and cDNA was synthesized with SuperScript III First-Strand Synthesis SuperMix (Invitrogen, 18080400). RT-qPCR was performed with SYBR Green PCR Master Mix (Applied Biosystems, 4309155) and analyzed on an ABI 7500 Sequence Detection System, with gene expression levels normalized to Actin. For mitochondrial DNA quantification, DNA from c-Kit$^+$ cells was extracted using a lysis buffer (10 mM Tris-HCl pH7.5, 50 mM NaCl, 6.25 mM $MgCl_2$, 0.045% NP40, 0.45% Tween20, 1 mg/mL proteinase K) as previously described (1, 2). MtDNA quantification was performed by RT-qPCR, calculating the relative mtDNA (ND2) to nuclear DNA (Nme1) ratio using the ΔΔCt method (3). PCR primers were listed in Supplemental Table.

Acetyl-CoA levels, NAD/NADH ratio, and ATP measurements

Acetyl-CoA levels and the NAD/NADH ratio were measured in c-Kit^{+} cells, and ATP levels were quantified in sorted LT-HSCs, following the manufacturer's protocols. The measurements were performed using the PicoProbe Acetyl CoA Assay Kit (Abcam, ab87546), the NAD/NADH Assay Kit (Abcam, ab65348), and the ATP Determination Kit (Thermo Fisher Scientific, A22066).

Measurements of $\Delta\Psi$m, intracellular Ca^{2+}, mitochondrial Ca^{2+}, cell superoxide, and γH2A.X levels

$\Delta\Psi$m, mitochondrial superoxide, and mitochondrial mass were determined in HSCs according to the manufacturer's instruction using the MitoProbe TMRE Assay kit (Thermo Fisher Scientific, M20036). Intracellular Ca^{2+}, mitochondrial Ca^{2+}, mitochondrial superoxide, cell superoxide, and γH2A.X levels were determined using Fluo-4, AM (Thermo Fisher Scientific, F14201), Rhod-2, AM (Thermo Fisher Scientific, R1244), CellROX Deep Red (Invitrogen, C10422), and Alexa Fluor 647 mouse Anti-H2A.X (Biosciences, 51-9007683), respectively. Cells were stained with 1 μM Fluo-4, AM, 1 μM Rhod-2, AM, 5 μM CellROX Red, or 5 μM Anti-H2A.X for 30 minutes, and fluorescence intensity was measured using a flow cytometer.

Western blotting

To assess CPT1A expression, c-Kit^{+} cells were purified and lysed, and proteins were detected using an anti-CPT1A antibody (Proteintech, 15184-1-AP, 1:2000). For SDS-PAGE analysis and CPT1A expression following genotoxic stress, mitochondria were isolated from bone marrow (BM) cells. Equal amounts of mitochondrial protein were loaded onto the gel, and proteins detected using anti-Citrate Synthase (CST, 14309, 1:500) and anti-ACO2 (CST, 6571, 1:2000) antibodies which served as internal controls.

Etomoxir treatment *in vivo*

WT mice, aged six to eight weeks, were administered Etomoxir (MCE, HY-50202, 25 mg/kg) by intraperitoneal (i.p.) injection every other day. Etomoxir was diluted according to the manufacturer's protocol. After treatment, mice were harvested at 3 days, 5 days, 2 weeks, and 4 weeks. Enumeration of HSCs and cell cycle analyses were performed as described earlier using flow cytometry.

References

1. van der Burg M, et al. Standardization of DNA isolation from low cell numbers for chimerism analysis by PCR of short tandem repeats. *Leukemia*. 2011;25(9):1467–1470.

2. Mariana Justino de Almeida, et al. Dye-Independent Methods Reveal Elevated Mitochondrial

Mass in Hematopoietic Stem Cells. *Cell Stem Cell*. 2017;176(12):139–148.

3. Hayashi Y, et al. Pathobiological pseudohypoxia as a putative mechanism underlying myelodysplastic syndromes. *Cancer Discov*. 2018;8(11):1438–1457.

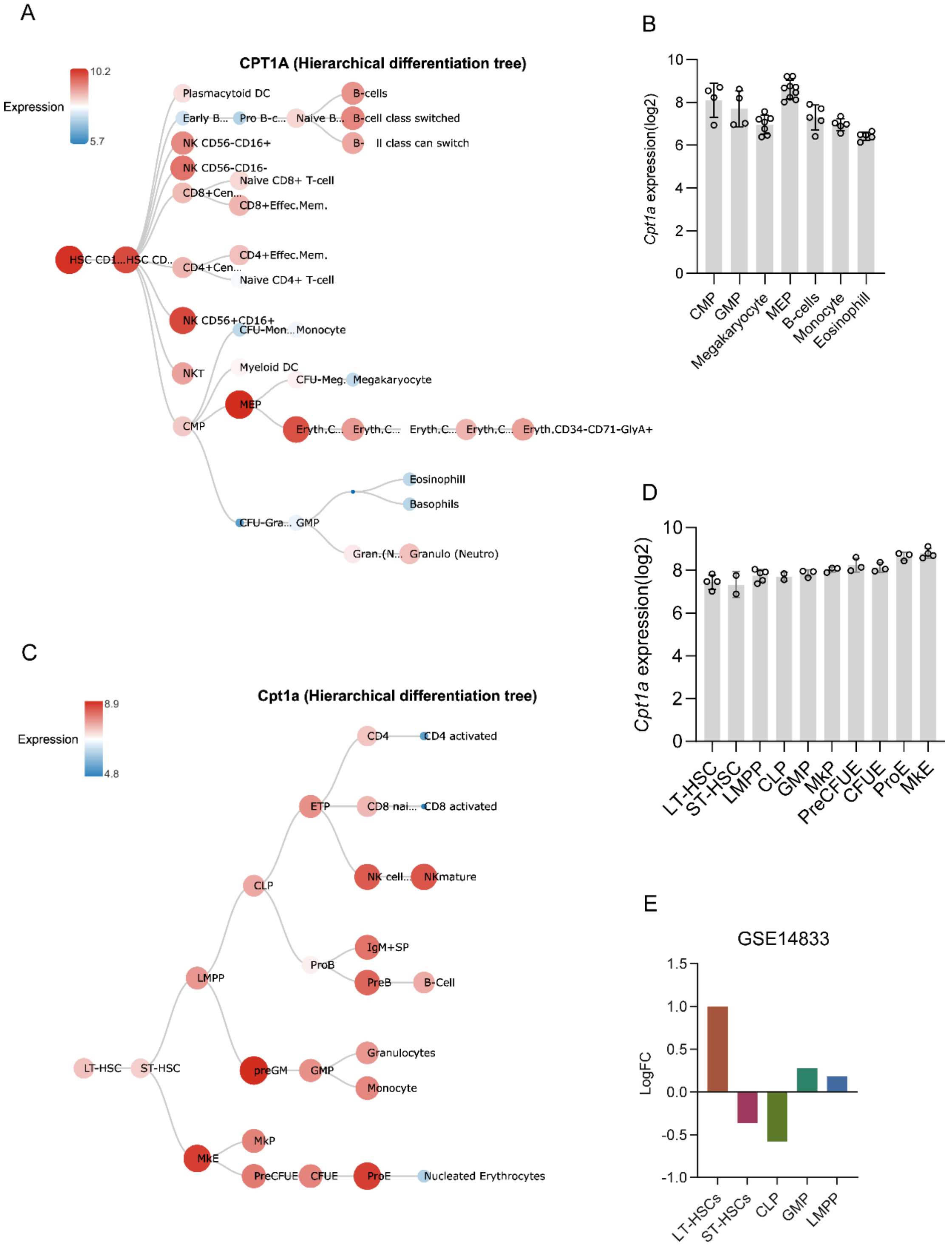

Figure S1. *Cpt1a* expression in human and mouse HSCs based on publicly-available datasets. **(A)** Expression of the *CPT1A* gene in the human HSC hierarchical differentiation tree sourced from

bloodspot.com. **(B)** Statistics of *CPT1A* expression in human normal HSCs. n = 4 or 9. **(C)** Expression of the *Cpt1a* gene in the mouse HSC hierarchical differentiation tree obtained from bloodspot.com. **(D)** Statistics of *Cpt1a* expression in normal mouse HSCs. n = 3 or 4. **(E)** Expression of the *Cpt1a* gene in mouse HSCs, as reported in GSE14833. n=1.

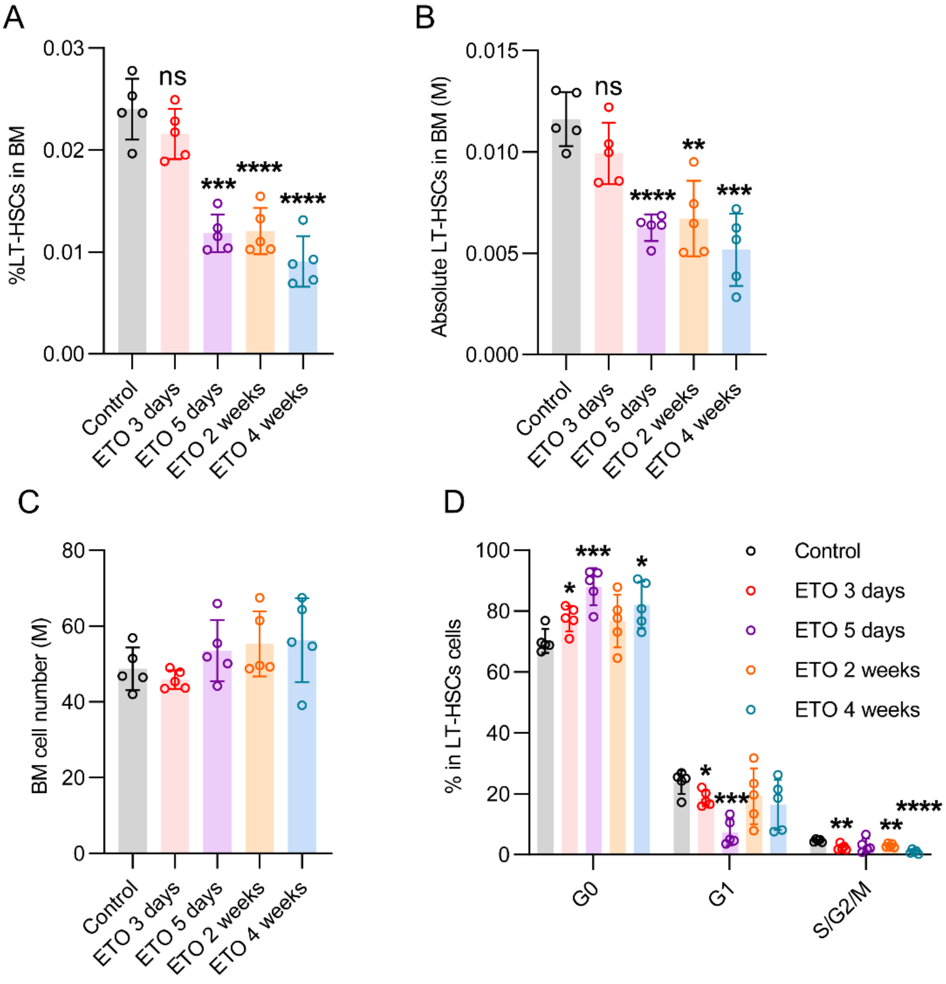

Figure S2. LT-HSC maintenance in WT mice treated with the CPT1A inhibitor Etomoxir (ETO). (A-B) Frequencies and absolute numbers of LT-HSCs in the BM of Etomoxir-treated mice at different time points. **(C)** Total BM cell numbers in mice treated with Etomoxir at different time points. **(D)** Frequencies of different cell cycle stages in LT-HSCs of Etomoxir-treated mice at different time points. n = 5 per group. Statistical significance was determined using Student *t* test. Data represent mean ± SEM. Statistical significance was determined using Student *t* test. ns, not significant, $*P < 0.05$, $**P < 0.01$, $***P < 0.001$, $****P < 0.0001$.

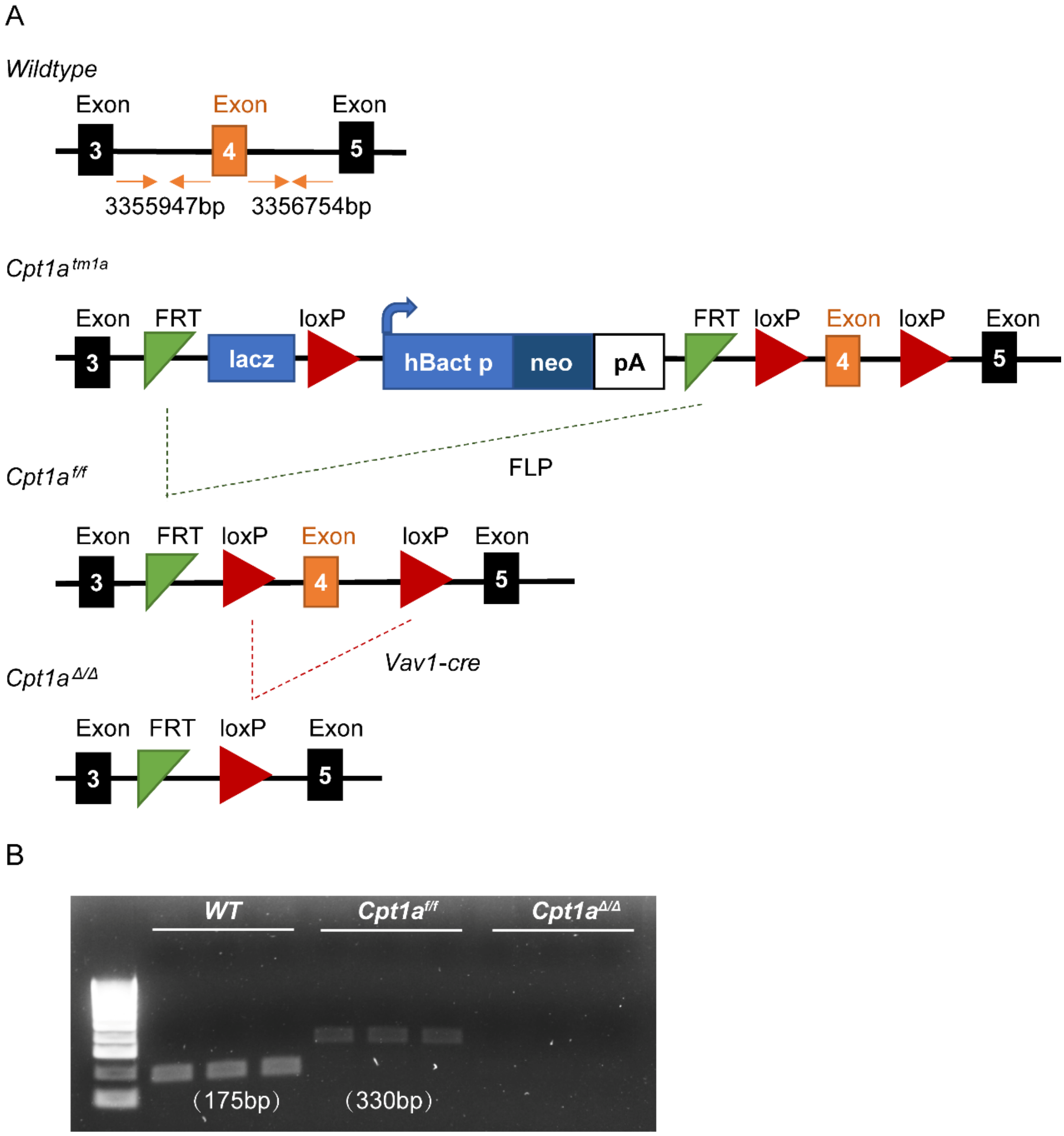

Figure S3. Strategy for generating a hematopoietic *Cpt1a* conditional knock-out mouse model. **(A)** Schematic for developing *Cpt1a*$^{\Delta/\Delta}$ mice by intercrossing *Cpt1a*$^{f/f}$ mice with *Vav1-cre*$^{+}$ mice. **(B)** Genotyping using c-Kit^{+} cells by genomic PCR.

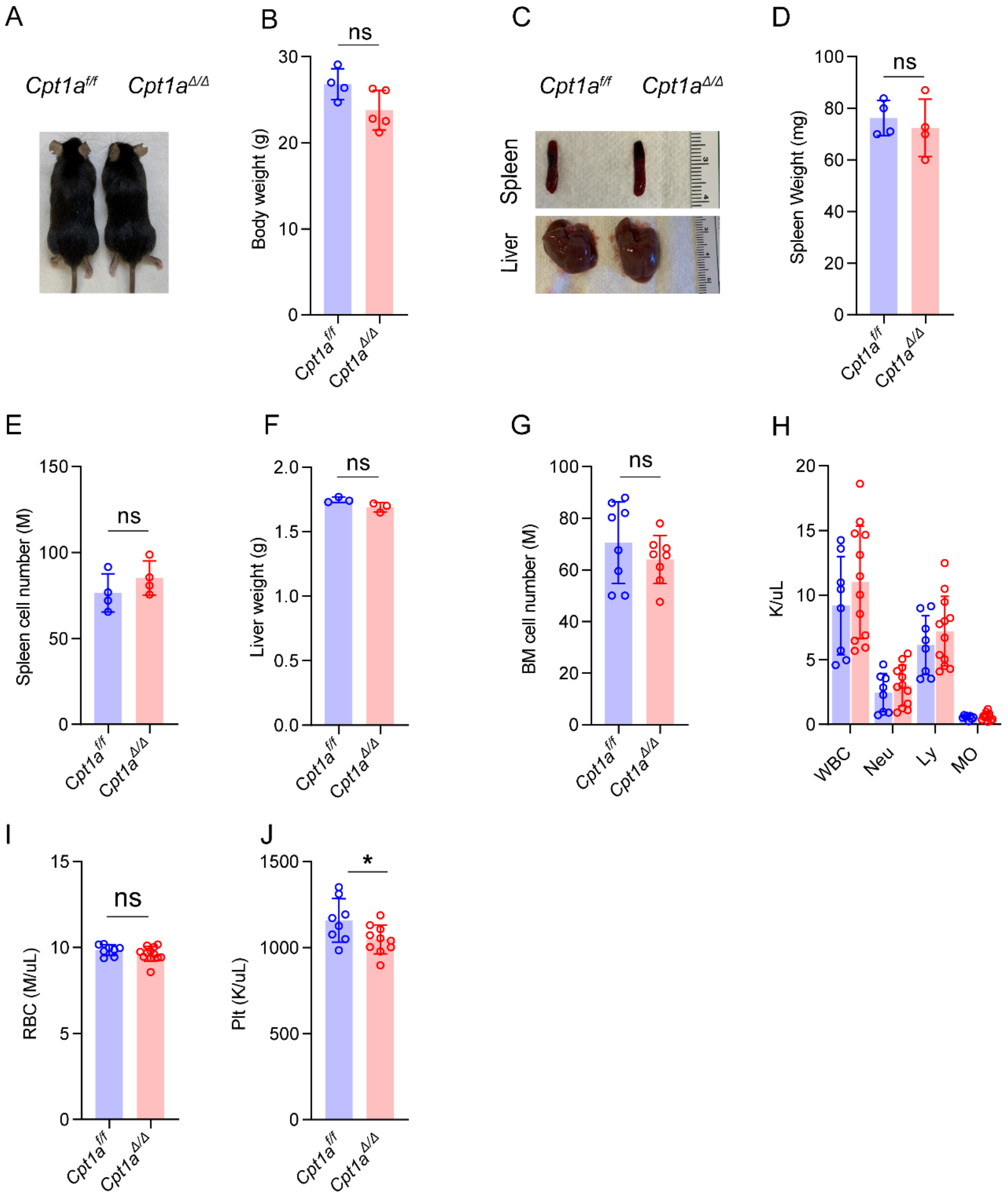

Figure S4 Phenotypic characterization of *Cpt1a*$^{\Delta/\Delta}$ mice. (A) Representative body photo. **(B)** Statistics of body weight. n = 4 or 5. **(C)** Upper: Representative photo of the spleen; Below: Representative photos of the liver. **(D)** Statistics of spleen weight. n = 4 per group. **(E)** Statistics of spleen cell numbers. n = 4 per group. **(F)** Statistics of liver weight. n = 3 per group. **(G)** Statistics of bone marrow cell numbers. n = 8 per group. **(H)** Complete blood count: white blood cells (WBC), neutrophils (Neu), lymphoid (Ly), monocytes (MO). n = 8 or 12. **(I)** Red blood cell counts. n =8 or 12. **(J)** Platelet cell counts. n = 8 or 10. Data represent mean ± SEM. Statistical significance was determined using Student *t* test. ns, not significant, *$P < 0.05$.

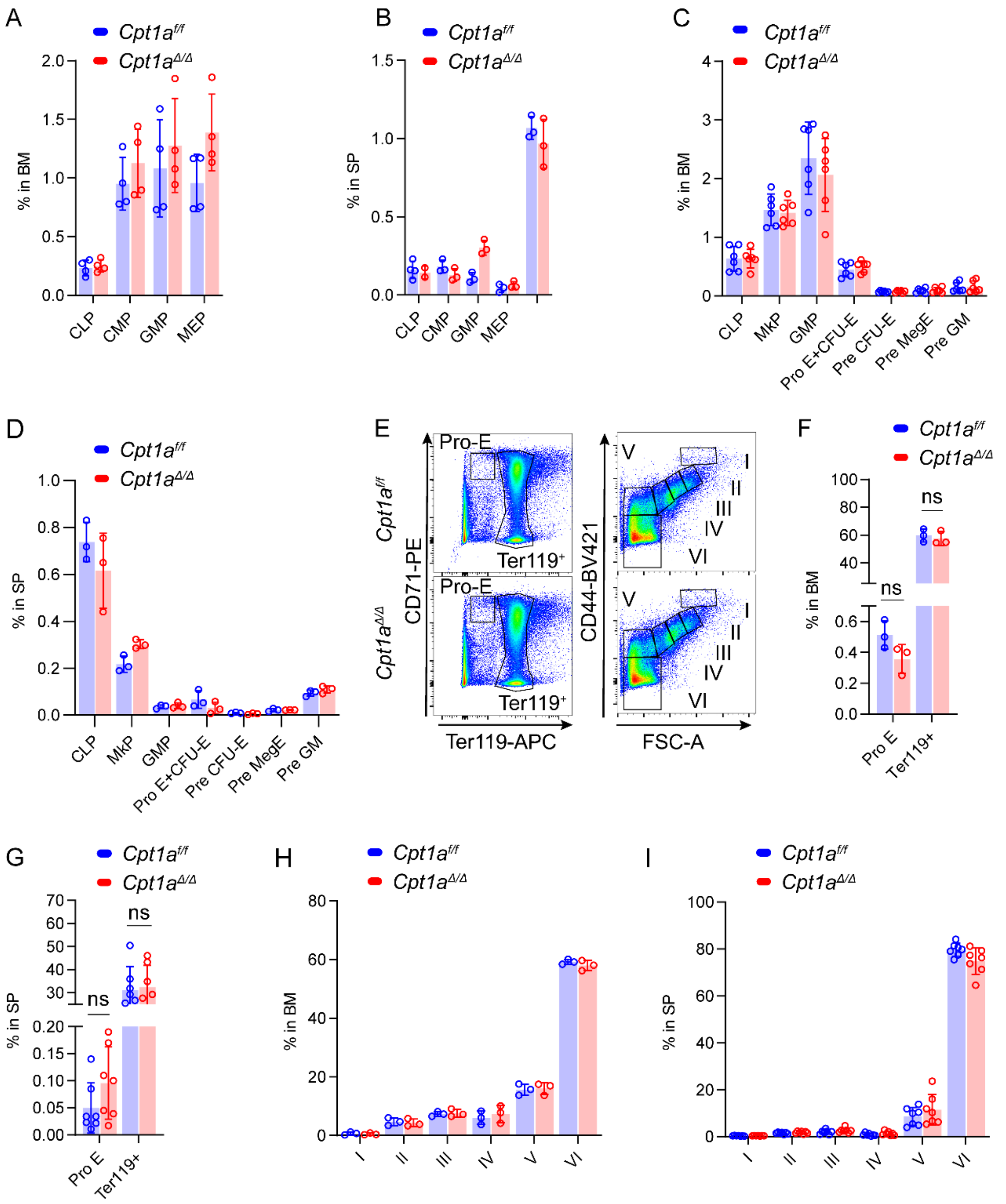

Figure S5. Phenotypic characterization of $Cpt1a^{\Delta/\Delta}$ HSPCs. **(A-B)** Frequencies of HSPCs in the BM and spleen. n = 3 or 4. **(C-D)** Frequencies of HSPCs in the BM and spleen. n = 6 or 3. **(E)** Representative flow cytometric analysis of terminal erythroid differentiation populations: proerythroblasts (I), basophilic erythroblasts (II), polychromatic erythroblasts (III), orthochromatic erythroblasts (IV), reticulocytes (V), and mature RBCs (VI) in BM. **(F-G)** Frequencies of Pro E and Ter119^{+} population blasts in the BM and spleen. n =3 or 7. **(H-I)** Frequencies of erythroid blasts in BM and spleen. n = 3 or 7. Data represent mean ± SEM. Statistical significance was determined using Student *t* test. ns, not significant.

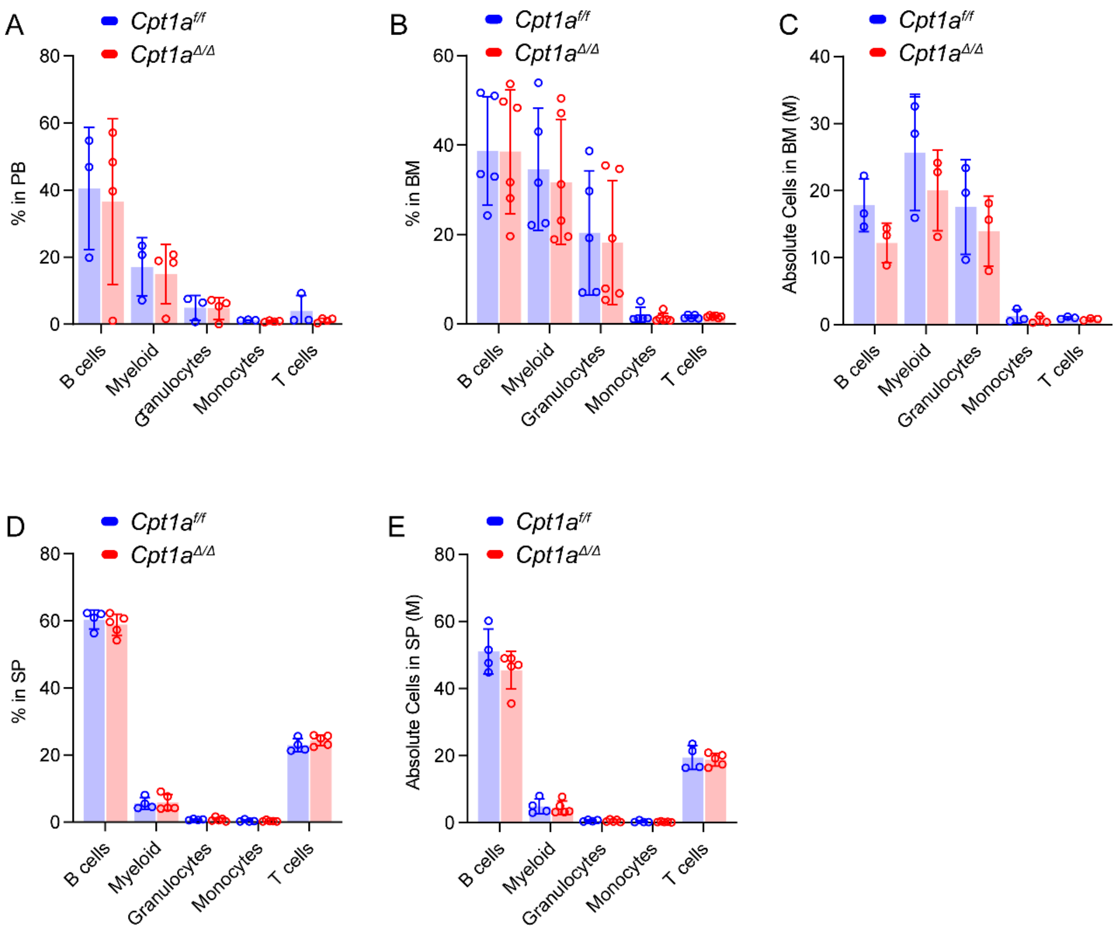

Figure S6. Characterization of multiple hematopoietic lineages of *Cpt1a*$^{Δ/Δ}$ mice. (A) Frequencies of indicated mature lineage cells in the PB. n = 4 per group. **(B-C)** Frequencies and absolute number of the indicated mature lineage cells in the BM. n = 5 or 3. **(D-E)** Frequencies and absolute number of the indicated mature lineage cells in the spleen. n = 4 or 5. Data represent mean ± SEM. Statistical significance was determined using Student *t* test.

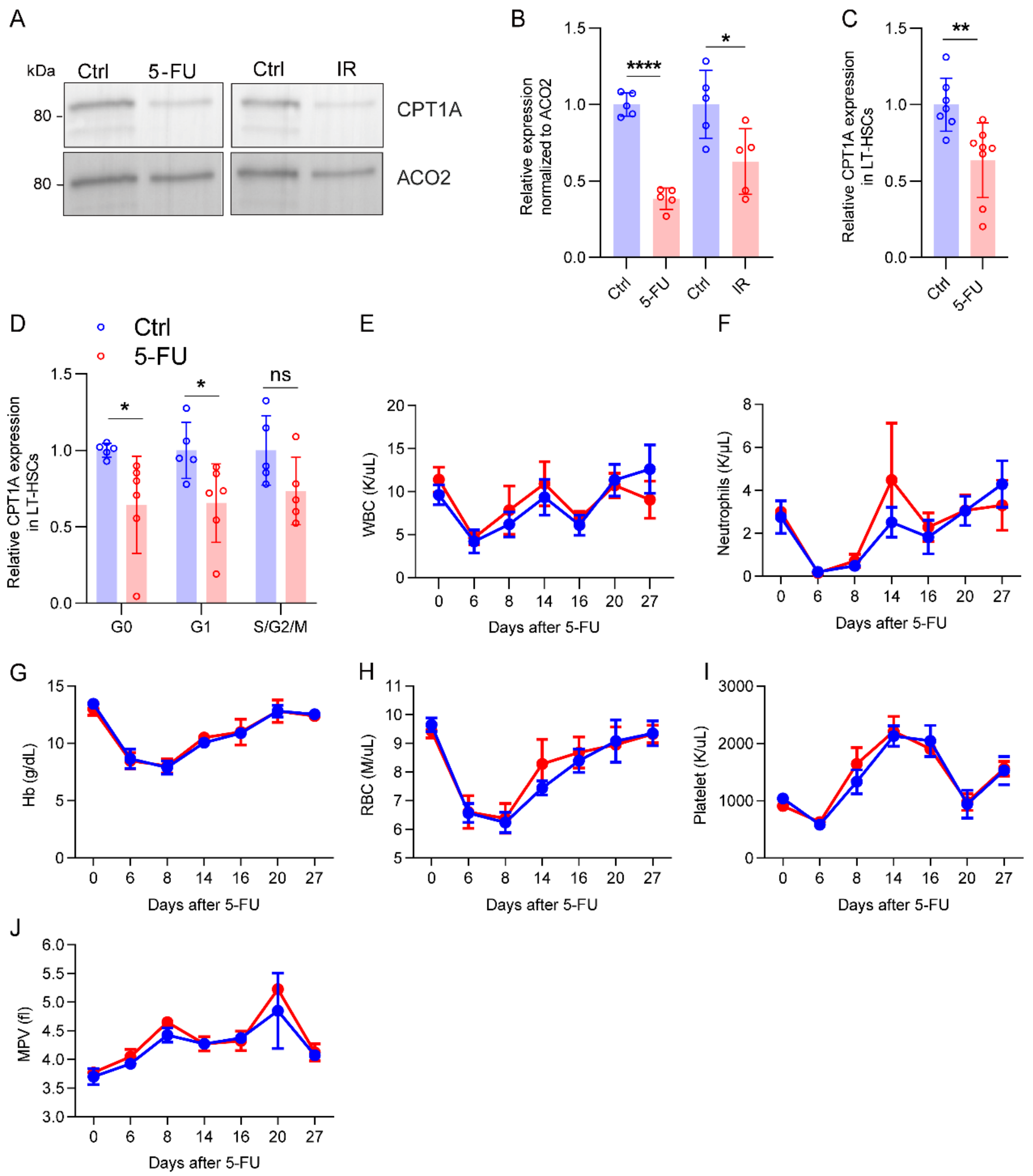

Figure S7. Complete blood cell counts after 5-FU treatment. (A) Western blotting analysis of the expression of CPT1A in Control (Ctrl), 5-FU treated mice, and γ-irradiation treated mice. ACO2 as an internal control. Representative data of five independent experiments is shown. The CPT1A and ACO2 panels were derived from the same membrane by stripping and reprobing. **(B)** Statistics of the expression of CPT1A normalized to ACO2 in Ctrl, 5-FU treated mice, and γ-irradiation treated mice. n = 5 per group. **(C)** Relative expression of CPT1A in LT-HSCs measured by flow cytometry. n = 7 or 8. **(D)** Relative expression of CPT1A in different cell cycle stages in LT-HSCs, as measured by flow cytometry. n = 5 per group. **(E-J)** Serial complete blood cell counts after treatment with 5-FU. WBC: white blood cell, Hb: Hemoglobin; RBC: red blood cell; MPV: Mean Platelet Volume. n = 4 per group. Data represent mean ± SEM. Statistical significance was determined using Student *t* test. ns, not significant, *$P < 0.05$, **$P < 0.01$, ****$P < 0.0001$.

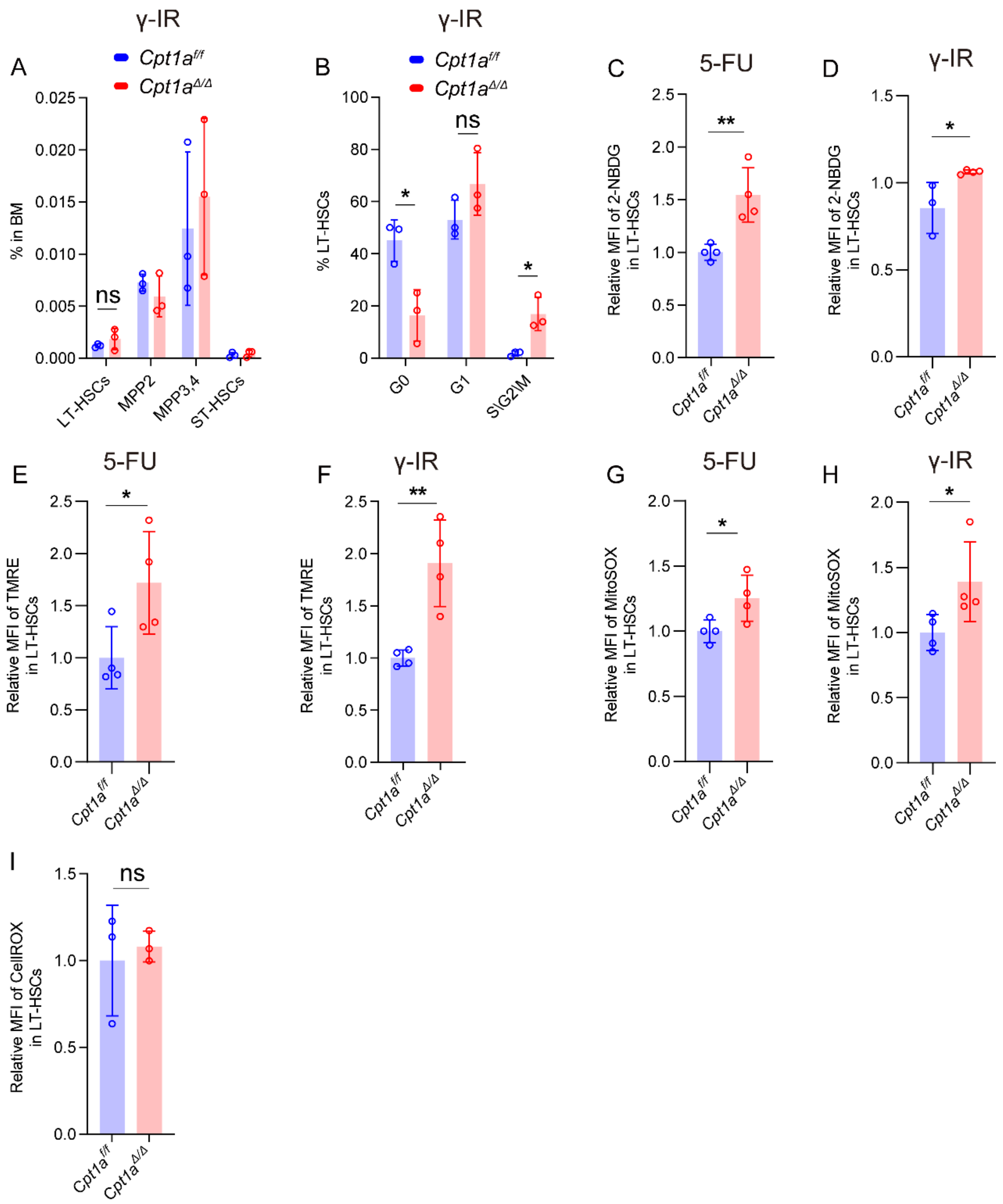

Figure S8. HSC phenotypes, and mitochondrial activity in *Cpt1a*$^{\Delta/\Delta}$ HSCs under genotoxic stress. (**A**) Frequencies of HSCs in the BM of mice treated with γ-irradiation. n = 3 per group. **(B)** Frequencies of different cell cycle stages in LT-HSCs of mice treated with γ-irradiation. n = 3 per group. **(C-D)** Relative glucose uptake levels measured using 2-NBDG in LT-HSCs of mice treated with 5-FU and γ-irradiation. n = 4 or 3. **(E-F)** Relative MFI levels of TMRE in LT-HSCs of mice treated with 5-FU and γ-irradiation. n = 4 per group. **(G-H)** Relative levels of mitochondrial ROS levels in LT-HSCs from mice treated with 5-FU and γ-irradiation, as detected by staining with MitoSOX. n = 4 per group. **(I)** Relative levels of cellular ROS staining in LT-HSCs, detected by staining with CellROX red. n = 3 per group. Data represent mean ± SEM. Statistical significance was determined using Student *t* test. ns, not significant, *$P < 0.05$, **$P < 0.01$.

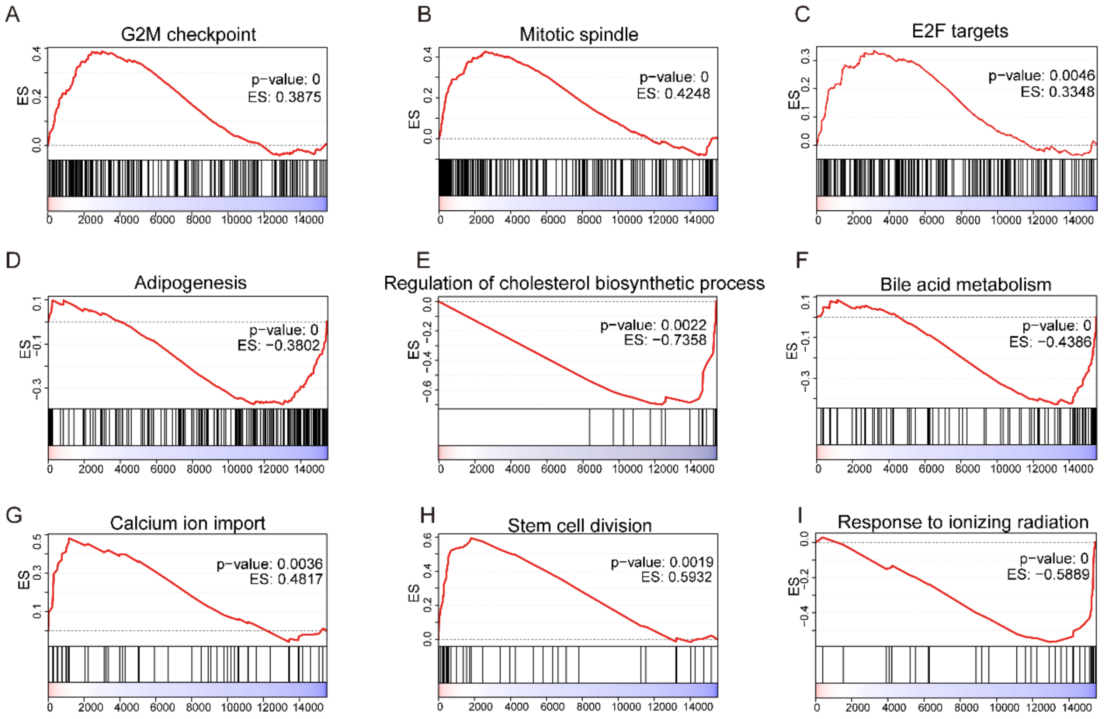

Figure S9. GSEA analysis. (A) G2/M checkpoint pathway. **(B)** Mitotic spindle pathway. **(C)** E2F targets pathway. **(D)** Adipogenesis pathway. **(E)** Regulation of cholesterol biosynthetic process pathway. **(F)** Bile acid metabolism pathway. **(G)** Calcium ion import pathway. **(H)** Stem cell division pathway. **(I)** Response to ionizing radiation pathway.
