# Supplementary material for: Loss of Cpt1a results in elevated glucose-fueled mitochondrial oxidative phosphorylation and defective hematopoietic stem cells
Source: J Clin Invest. 2025 Jan 9;135(5):e184069. doi: 10.1172/JCI184069 (PMC11870731; doi:10.1172/JCI184069)
Supplement: Unedited blot and gel images [file jci-135-184069-s218.pdf]

Figure 1B

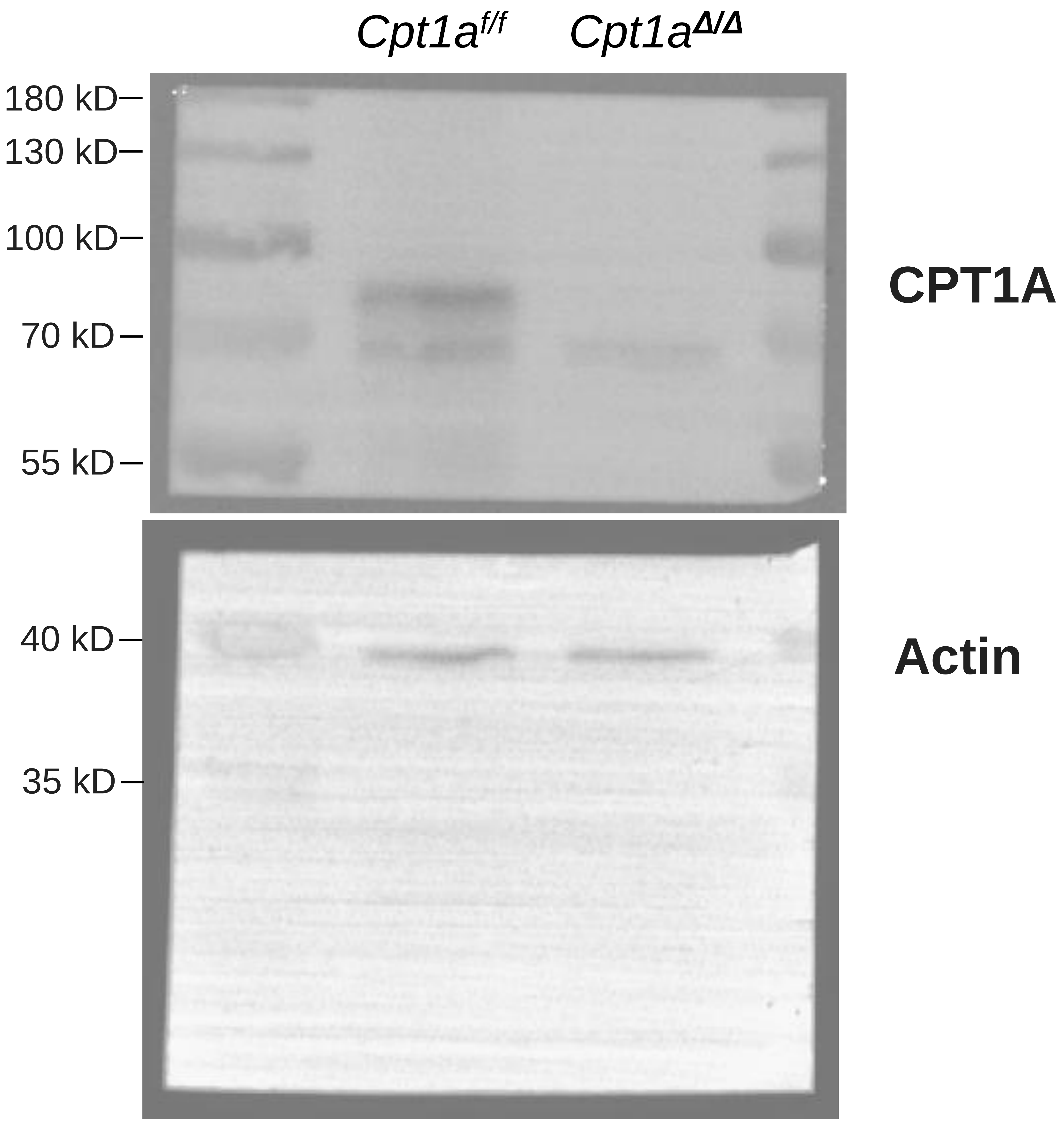

Cpt1a$^{f/f}$
Cpt1a$^{\Delta/\Delta}$
180 kD
130 kD
100 kD
70 kD
55 kD
CPT1A
40 kD
35 kD
Actin

Figure 4D

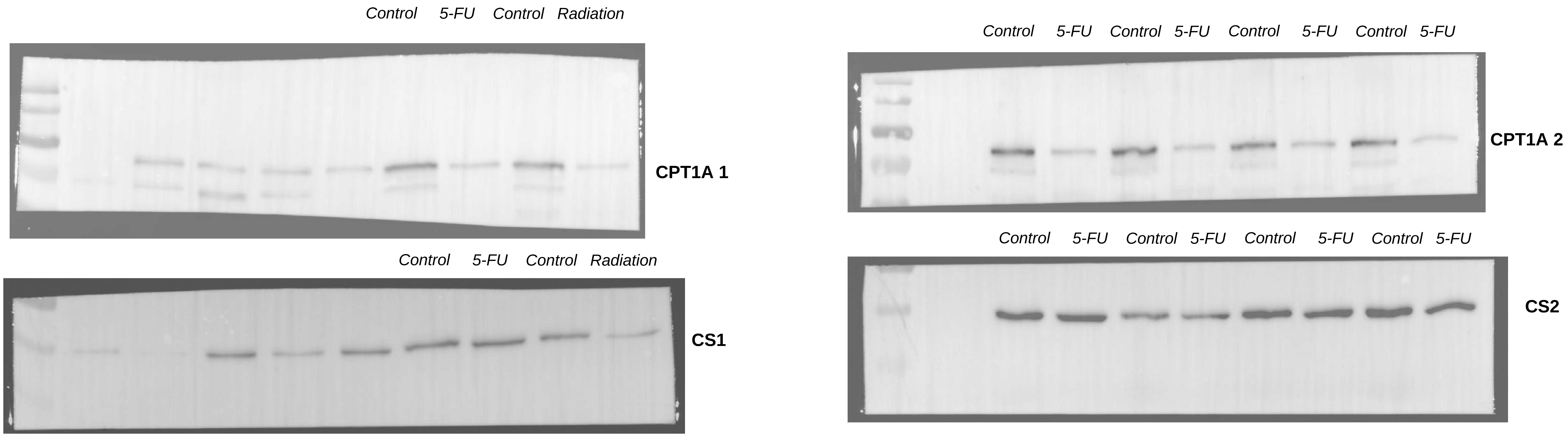
Control 5-FU Control Radiation
CPT1A 1
Control 5-FU Control Radiation
CS1
Control 5-FU Control 5-FU Control 5-FU Control 5-FU
CPT1A 2
Control 5-FU Control 5-FU Control 5-FU Control 5-FU
CS2

Figure 4D

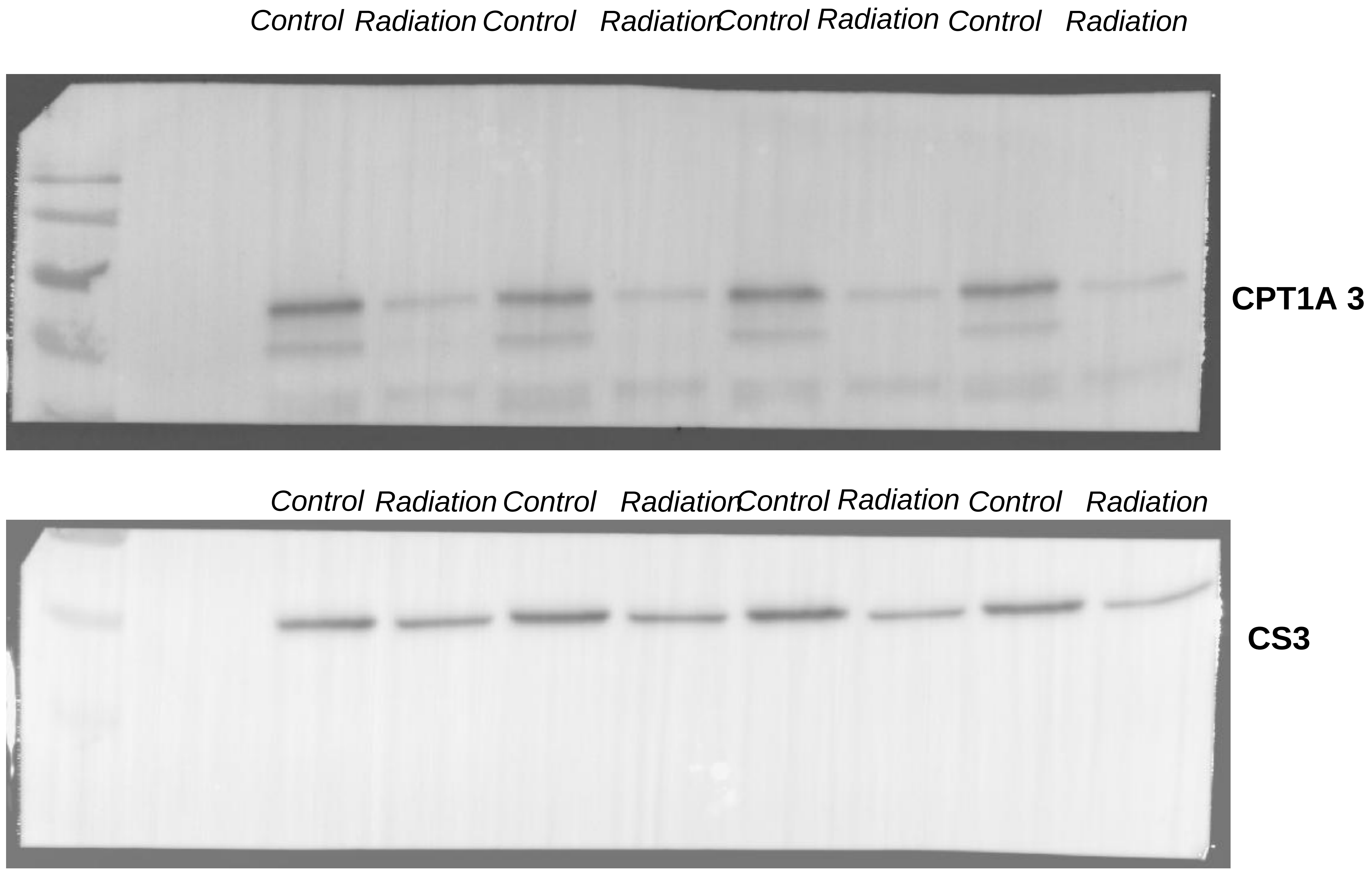
Control
Radiation
Control
Radiation
Control
Radiation
Control
Radiation
CPT1A 3
Control
Radiation
Control
Radiation
Control
Radiation
Control
Radiation
CS3

Figure 6A

Replicate 1

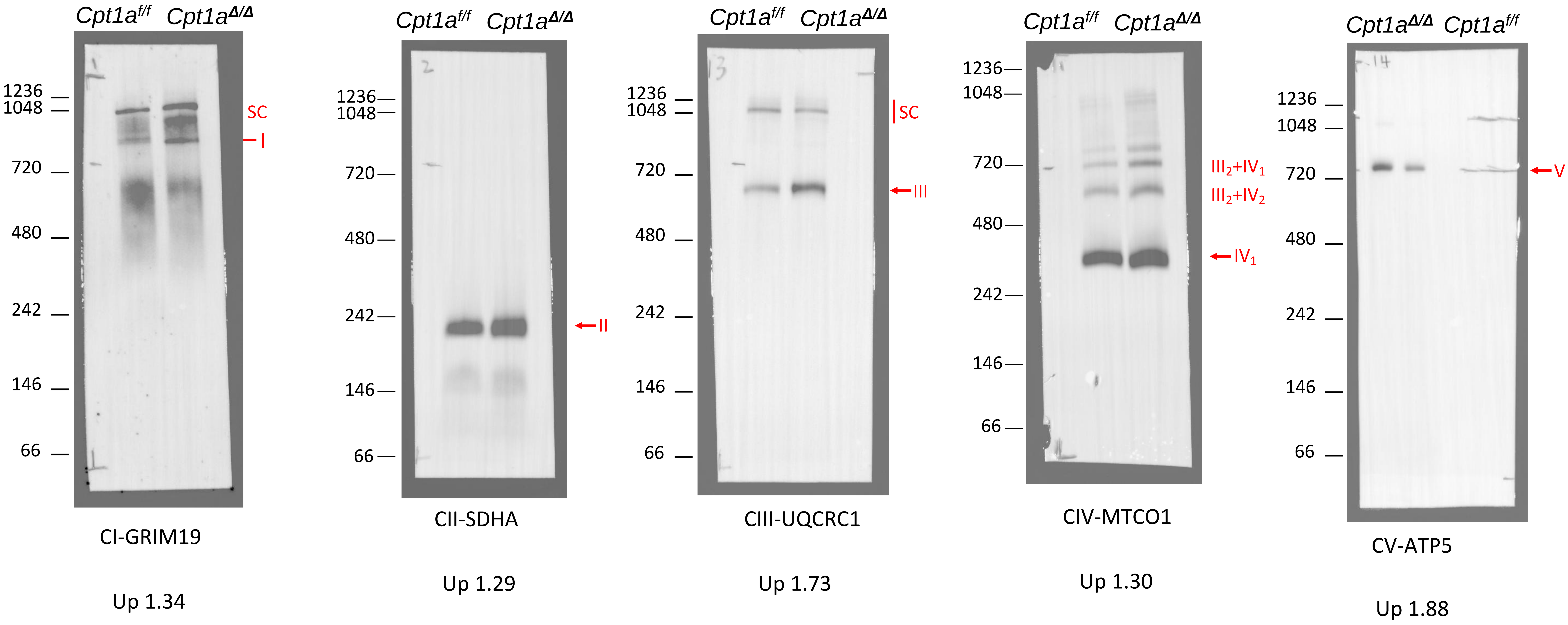

Replicate 2

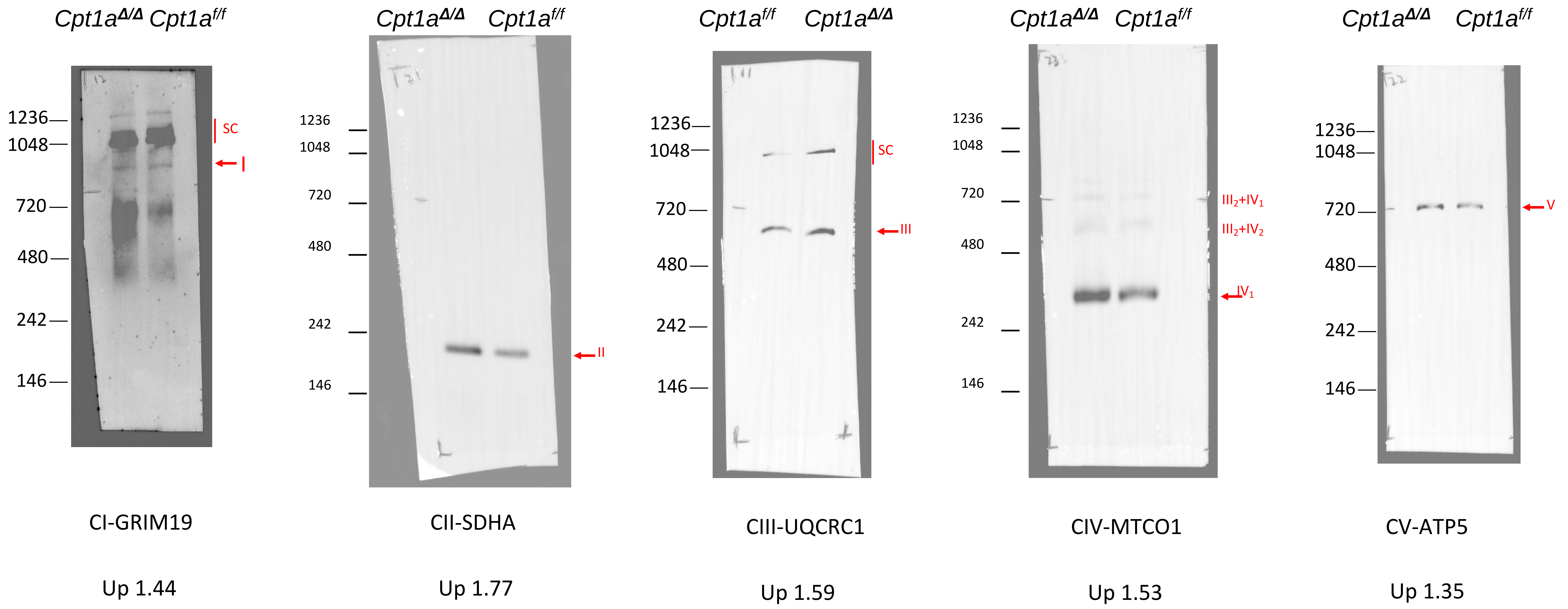
Cpt1aΔ/Δ Cpt1af/f
1236
1048
720
480
242
146
SC
I
CI-GRIM19
Up 1.44
Cpt1aΔ/Δ Cpt1af/f
1236
1048
720
480
242
146
II
CII-SDHA
Up 1.77
Cpt1af/f Cpt1aΔ/Δ
1236
1048
720
480
242
146
SC
III
CIII-UQCRC1
Up 1.59
Cpt1aΔ/Δ Cpt1af/f
1236
1048
720
480
242
146
III2+IV1
III2+IV2
IV1
CIV-MTCO1
Up 1.53
Cpt1aΔ/Δ Cpt1af/f
1236
1048
720
480
242
146
V
CV-ATP5
Up 1.35

Replicate 3

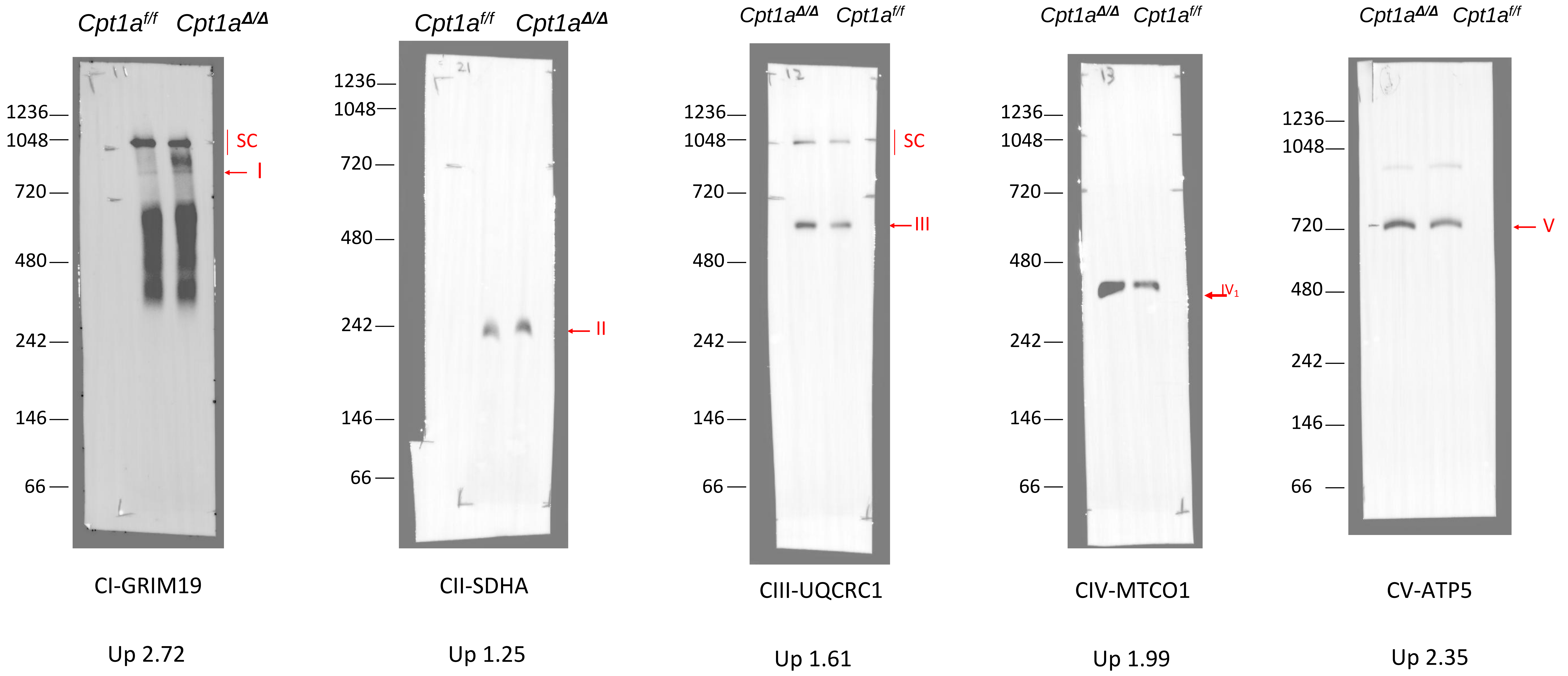

Cpt1a f/f
Cpt1a Δ/Δ
1236
1048
720
480
242
146
66
SC
I
CI-GRIM19
Up 2.72
Cpt1a f/f
Cpt1a Δ/Δ
1236
1048
720
480
242
146
66
II
CII-SDHA
Up 1.25
Cpt1a Δ/Δ
Cpt1a f/f
1236
1048
720
480
242
146
66
SC
III
CIII-UQCRC1
Up 1.61
Cpt1a Δ/Δ
Cpt1a f/f
1236
1048
720
480
242
146
66
IV1
CIV-MTCO1
Up 1.99
Cpt1a Δ/Δ
Cpt1a f/f
1236
1048
720
480
242
146
66
V
CV-ATP5
Up 2.35

Figure 7A

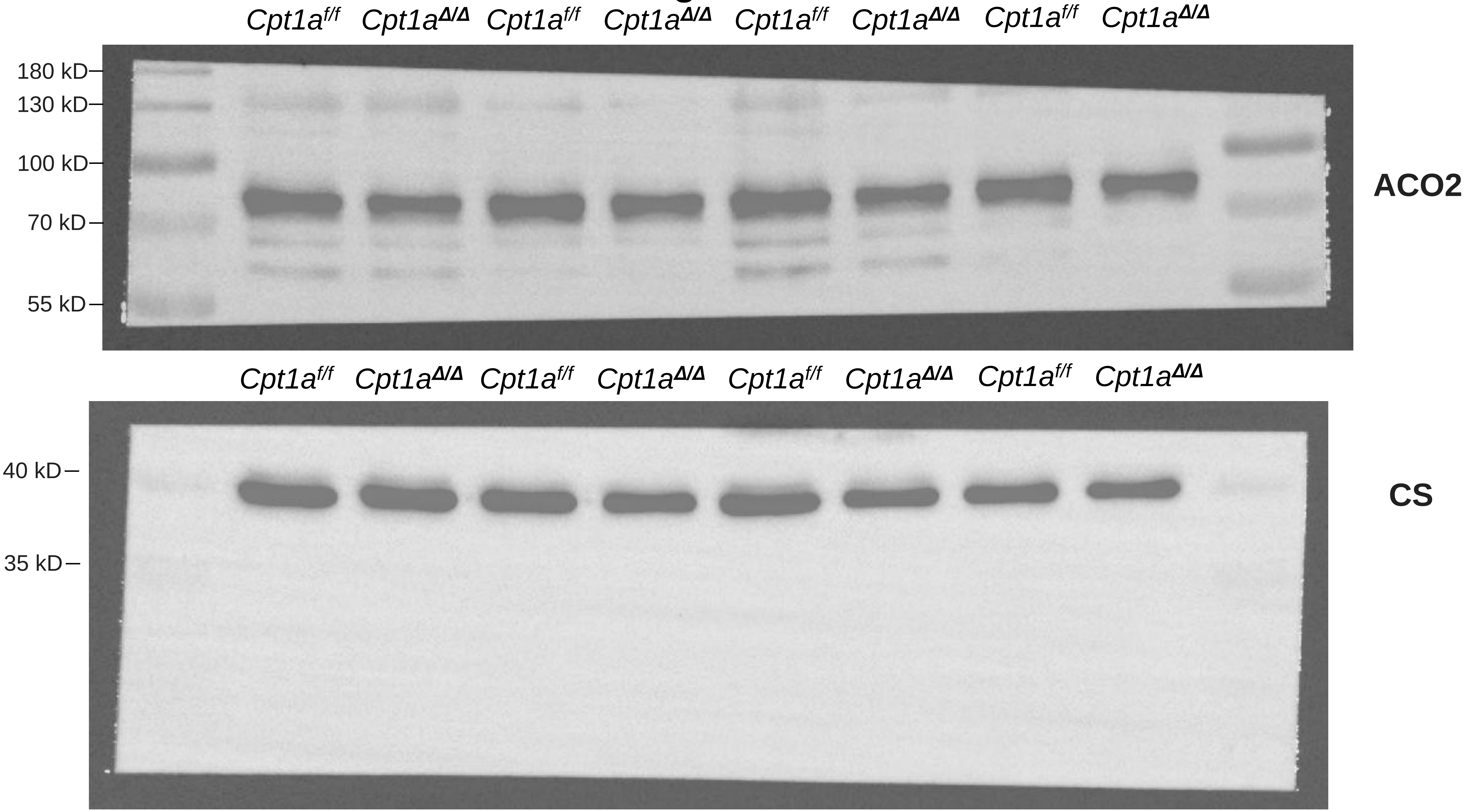

$Cpt1a^{f/f}$ $Cpt1a^{\Delta/\Delta}$ $Cpt1a^{f/f}$ $Cpt1a^{\Delta/\Delta}$ $Cpt1a^{f/f}$ $Cpt1a^{\Delta/\Delta}$ $Cpt1a^{f/f}$ $Cpt1a^{\Delta/\Delta}$
180 kD
130 kD
100 kD
70 kD
55 kD
ACO2
$Cpt1a^{f/f}$ $Cpt1a^{\Delta/\Delta}$ $Cpt1a^{f/f}$ $Cpt1a^{\Delta/\Delta}$ $Cpt1a^{f/f}$ $Cpt1a^{\Delta/\Delta}$ $Cpt1a^{f/f}$ $Cpt1a^{\Delta/\Delta}$
40 kD
35 kD
CS

Figure S7A

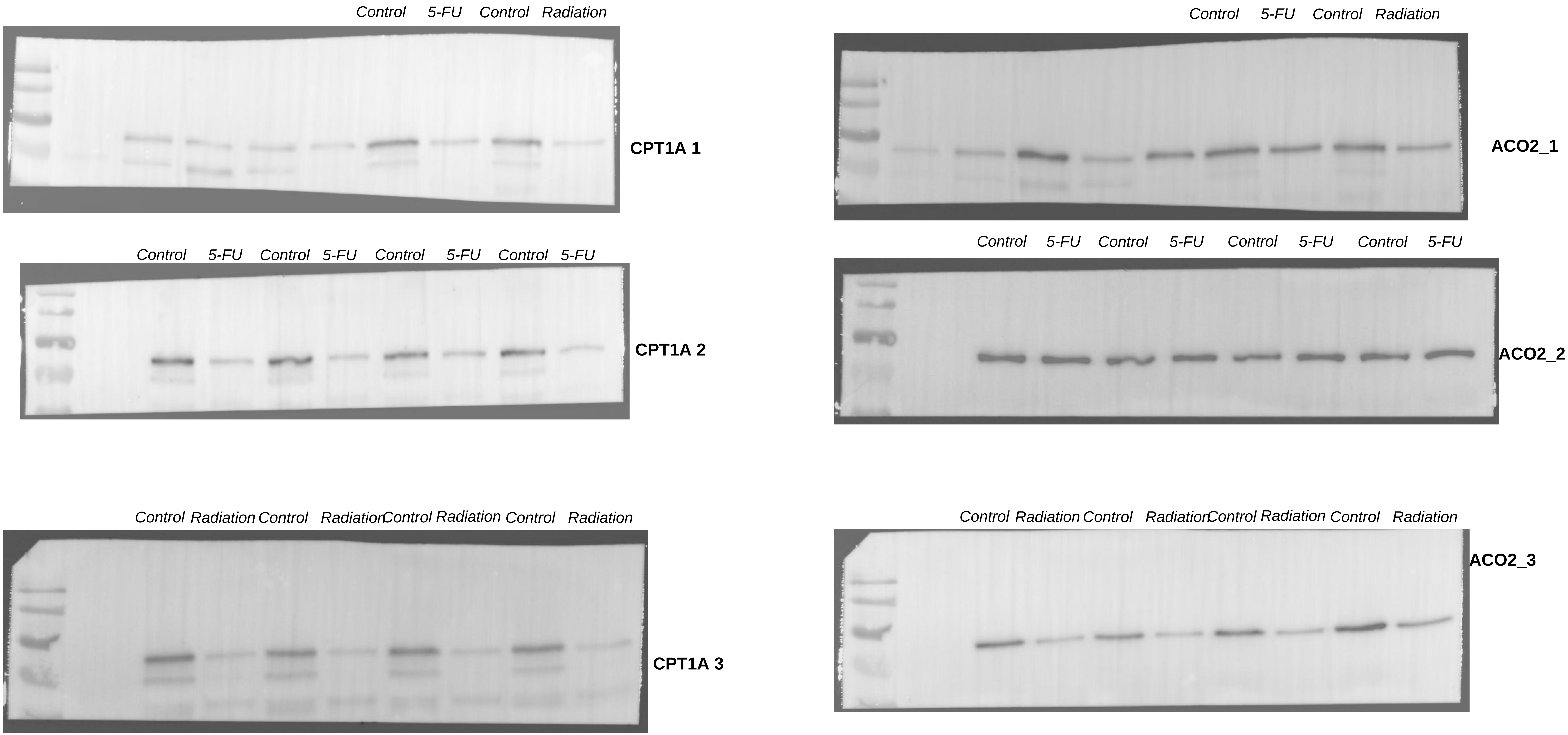
Control 5-FU Control Radiation
CPT1A 1
Control 5-FU Control Radiation
ACO2_1
Control 5-FU Control 5-FU Control 5-FU Control 5-FU
CPT1A 2
Control 5-FU Control 5-FU Control 5-FU Control 5-FU
ACO2_2
Control Radiation Control Radiation Control Radiation Control Radiation
CPT1A 3
Control Radiation Control Radiation Control Radiation Control Radiation
ACO2_3
